# Basidiomycota strains as whole-cell biocatalysts for the synthesis of high-value natural benzaldehydes

**DOI:** 10.1007/s00253-023-12872-y

**Published:** 2024-01-11

**Authors:** Stefano Serra, Stefano Marzorati, Ewa Szczepańska, Tomasz Strzała, Filip Boratyński

**Affiliations:** 1 Consiglio Nazionale delle Ricerche (C.N.R.), Istituto di Scienze e Tecnologie Chimiche, Via Mancinelli 7, 20131 Milan, Italy; 2 Department of Food Chemistry and Biocatalysis, Wrocław University of Environmental and Life Sciences, Norwida 25, 50-375 Wrocław, Poland; 3 Department of Genetics, Wroclaw University of Environmental and Life Sciences, Ul. Kożuchowska 7, 51-631 Wrocław, Poland

**Keywords:** Natural flavours, Vanillin, Veratraldehyde, Piperonal, Biotransformations, Basidiomycetes

## Abstract

**Abstract:**

Substituted benzaldehydes are the most commonly used natural-occurring flavours in the world. The consumer’s preference for ‘natural or organic’ aromas has increased the request for flavours possessing the ‘natural’ status. The resulting shortage of aromatic aldehydes of extractive origin, such as vanillin, veratraldehyde and piperonal, can be offset by developing a new biotechnological synthesis method. Here, we report a study on the microbiological reduction of five natural benzoic acid derivatives, namely *p*-anisic, vanillic, veratric, piperonylic and eudesmic acids, to produce the corresponding fragrant aldehydes. We found that different Basidiomycota strains can efficiently perform this transformation, with good chemical selectivity and tolerance to the toxicity of substrates and products. Besides confirming the carboxylic acid reductase activity of the already studied fungi *Pycnoporus cinnabarinus*, we discovered that other species such as *Pleurotus eryngii*, *Pleurotus sapidus* and *Laetiporus sulphureus* as well as the non-ligninolytic fungi *Lepista nuda* are valuable microorganisms for the synthesis of anisaldehyde, vanillin, veratraldehyde, piperonal and 3,4,5-trimethoxybenzaldehyde from the corresponding acids. According to our findings, we propose a reliable process for the preparation of the above-mentioned aldehydes, in natural form.

**Key points:**

• *Fragrant benzaldehydes were obtained by biotransformation.*

• *Basidiomycota strains reduced substituted benzoic acid to the corresponding aldehydes.*

• *Anisaldehyde, vanillin, veratraldehyde, piperonal and 3,4,5-trimethoxybenzaldehyde were prepared in natural form.*

**Graphical Abstract:**

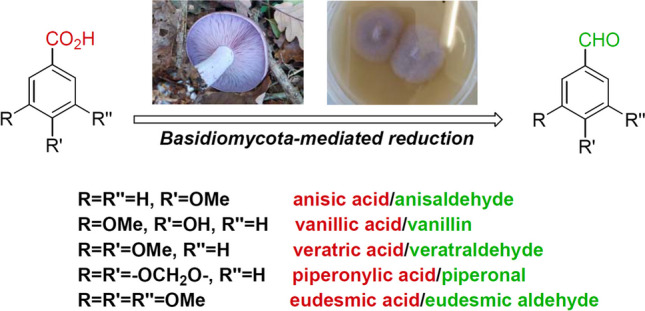

**Supplementary Information:**

The online version contains supplementary material available at 10.1007/s00253-023-12872-y.

## Introduction

**Fig. 1:**
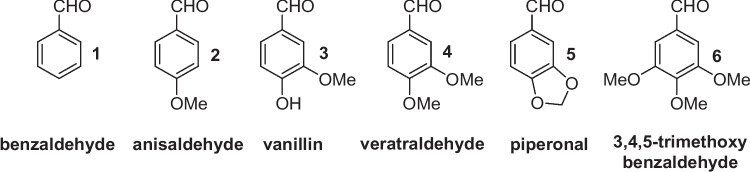
Some relevant natural benzaldehydes of interest in flavours and fragrance field

Substituted benzaldehydes of phenylpropanoid origin are the natural products of utmost industrial importance (Vogt 2010; Surburg and Panten 2016). These compounds have been widely employed for food flavouring. Their extraction from the natural sources or chemical synthesis can be considered the fundamental processes in the flavours and fragrances field. Benzaldehyde (**1**), anisaldehyde (**2**), vanillin (**3**), veratraldehyde (**4**) and piperonal (**5**) (Fig. 1) are considered the most important aromatic compounds in this aldehyde class (Burdock 2010), and their production has steadily increased over the years. The structurally related 3,4,5-trimethoxybenzaldehyde (**6**), also known as eudesmic aldehyde, is a natural-occurring compound, occasionally used as flavour ingredient.

**Fig. 2:**
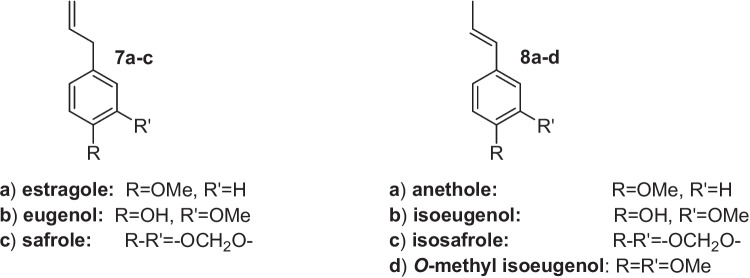
Natural propenylbenzenes (**7a–c**, **8a–d**)

Furthermore, modern consumers have developed an increasing ecological sensitivity, supporting the choice of environmentally friendly processes and the preference for ‘natural’ or ‘organic’ products, thus developing a new market for flavours of biotechnological origin. Since the flavours possessing the ‘natural’ status are usually hundreds of times more expensive than their synthetic counterparts, any new procedures that provide these compounds in their high-value form can be very profitable. According to the European (European Parliament 2008) and USA (U.S. Food & Drugs. Administration 2018) legislation, the biotransformation of a natural precursor is a ‘natural method’ of synthesis (Serra et al. 2005). Therefore, a number of biotechnological processes are currently employed to produce high-value natural benzaldehydes, especially for the compounds in which extraction from natural sources cannot fulfil the market requirements. For example, benzaldehyde (**1**) is prepared by retro-aldol reaction of natural cinnamaldehyde or enzyme-catalysed hydrolysis of amygdalin (Brenna et al. 2016). Natural vanillin (**3**) is prepared by microbial biotransformation of ferulic acid, eugenol or isoeugenol (Paul et al. 2021), whereas piperonal (**5**) can be obtained by biotransformation of isosafrole (Wen et al. 2019) or lipoxygenase-mediate oxidation of piperine (Krahe et al. 2021). Other aldehydes, such as anisaldehyde (**2**) and veratraldehyde (**4**), are available by extraction from natural sources and biotransformation of natural precursors. Overall, these flavours lack a common synthetic approach. Moreover, the processes affording vanillin (**3**) and piperonal (**5**) are not able to satisfy the commercial request of these aldehydes, in their natural form (Bomgardner 2016). For these reasons, a number of new studies on the biocatalysed synthesis of the aforementioned flavours have been published recently. Interestingly, a possible common approach to obtain aldehydes **2–5** can arise from the microbial or enzymatic transformation of the 2-propenylbenzene derivatives **7a–c** and 1-propenylbenzene **8a–d** (Xu et al. 2007). These phenylpropanoids (Fig. 2) are cheap and affordable flavours, usually obtained by physical extraction from plant essential oils.

**Fig. 3:**
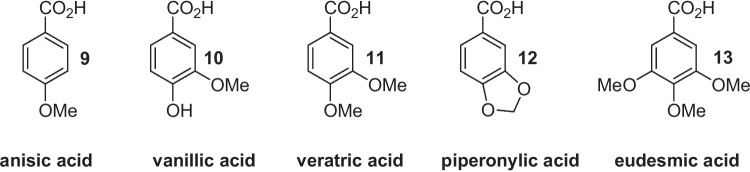
Benzoic acid derivatives obtained by propenylbenzene biotransformation (**9–12**) or by extraction from natural sources (**13**)

Although propenylbenzenes are toxic for most microorganisms, few fungal and bacterial strains can transform them in the corresponding substituted benzaldehydes or substituted benzoic acids. These findings have prompted many studies to elucidate the metabolic pathways involved in this kind of biotransformation. Recently, the use of genetic engineering techniques and the identification of the oxygenases involved in the propenylbenzene oxidation process (Ryu et al. 2010) allowed the development of new methods for the production of aldehydes **2–5** (Wang et al. 2021; Paul et al. 2021; Wen et al. 2019). The latter aldehydes are toxic for the transforming microorganisms, which usually do not stop the oxidation reactions, producing and accumulating the corresponding benzoic acid derivatives **9–12** (Fig. 3).

Therefore, the latter acids, in natural form, are now easily available thanks to the new processes of propenylbenzene biotransformation (Han et al. 2012). According to all these findings, we envisaged that acids **9–13** could be the proper natural precursors for the preparation of aldehydes **2–6**, respectively. Indeed, exploiting the catalytic activity of the carboxylic acid reductases (CARs), different bacterial and fungal strains can reduce the carboxylic acid functional group (Gahloth et al. 2020; Napora-Wijata et al. 2014). Nevertheless, the formed benzaldehydes are further reduced to the corresponding benzyl alcohol by the main part of these microorganisms. Usually, the latter reductive step proceeds with higher rate with the overall formation of benzyl alcohol only. Since some studies have shown that different Basidiomycetes can transform substituted benzoic acids into the corresponding aldehydes, we decided to investigate the microbial reduction of the acids **9–13** employing selected strains belonging to the Basidiomycota phylum. The screening performed by our research team on sixteen various strains, including white-rot, brown-rot, saprophytic and ectomycorrhizal species, allowed to select *Pleurotus eryngii*, *Pleurotus sapidus*, *Pycnoporus cinnabarinus*, *Lepista nuda* and *Laetiporus sulphureus* as valuable microorganisms for the synthesis of aldehydes **2–6**. Although some of these strains have already been employed to perform different chemical reactions, our study reports new insights in the field of natural flavour synthesis, pointing to the prospective employment of *Pleurotus*, *Pycnoporus* and *Lepista* strains for the production of anisaldehyde (**2**), vanillin (**3**), veratraldehyde (**4**), piperonal (**5**) and 3,4,5-trimethoxybenzaldehyde (**6**).

## Materials and methods

### Chemicals

All air and moisture sensitive reactions were carried out using dry solvents and under a static atmosphere of nitrogen. All solvents and reagents were of commercial quality and were purchased from Sigma-Aldrich (St. Louis, MO, USA). *p*-Anisic acid (**9**), vanillic acid (**10**), veratric acid (**11**), 3,4,5-trimethoxybenzoic acid (**13**), *p*-anisaldehyde (**2**), vanillin (**3**), veratric aldehyde (**4**), piperonal (**5**), casein peptone, peptone from soybean, yeast extract, meat extract, malt extract, sucrose, maltose, glucose, Amberlite® XAD1180N (20–60 mesh) and Na_2_SO_4_ were purchased from Sigma-Aldrich.

Piperonylic acid (**12**) was obtained by oxidation of piperonal (**5**) using silver oxide as oxidant (Pearl 1950). *p*-Anisyl alcohol (**14**), vanillyl alcohol (**15**), veratryl alcohol (**16**) and piperonyl alcohol (**17**) were prepared by NaBH_4_ reduction of the corresponding aldehydes. 3,4,5-Trimetoxybenzaldehyde (**6**) was obtained through MnO_2_ oxidation of 3,4,5-trimethoxybenzyl alcohol (**18**), which in turn was obtained by LiAlH_4_ reduction of 3,4,5-trimethoxybenzoic acid (**13**).

### Microorganisms and media

*Lepista nuda* (DSM 115118) and *Pleurotus eryngii* (DSM 114648) were isolated as axenic cultures in our laboratory, then identified through the ITS *r*DNA gene sequencing, using a previous described methodology (Mączka et al. 2018). After identification, DNA samples were deposited in the NCBI database under accession numbers ON764392 and ON764393 for *Lepista nuda* and *Pleurotus eryngii*, respectively, and finally deposited in the DSMZ GmbH collection (Braunschweig, Germany) under the collection number given in brackets.

*Poria placenta* (strains AM 36 and AM 38), *Inonotus radiatus* (AM 70), *Armillaria mellea* (strains AM 296 and AM 461), *Laetiporus sulphureus* (strains AM 498 and AM 515) and *Pholiota aurivella* (AM 522) were obtained from the collection of Wrocław University of Environmental and Life Sciences (Wrocław, Poland). *Pycnoporus cinnabarinus* (DSM 3022), *Pycnoporus cinnabarinus* (DSM 15225), *Pleurotus sapidus* (DSM 8266) and *Agrocybe aegerita* (DSM 22459) were purchased from DSMZ GmbH collection (Braunschweig, Germany). *Pycnoporus cinnabarinus* (CBS 353.63) and *Tricholoma terreum* (CBS 100138) were purchased from CBS-KNAW collection (Utrecht, The Netherlands).

The microorganisms were grown using the following media:

- Pre-grown medium 1 (PGM1): yeast extract (3 g/L), malt extract (3 g/L), peptone from soybean (3 g/L), casein peptone (2 g/L), glucose (10 g/L), potato extract (4 g/L), MgSO_4_·7H_2_O (1 g/L), CaCl_2_ (0.1 g/L) and trace elements solution (10 mL/L).
- Pre-grown medium 2 (PGM2): glucose (30 g/L) and peptone (10 g/L).
- Biotransformation medium (BM): maltose (20 g/L), yeast extract (1 g/L), diammonium tartrate (1.9 g/L), KH_2_PO_4_ (0.2 g/L), CaCl_2_ (20 mg/L), MgSO_4_·7H_2_O (0.5 g/L) and trace element solution (10 mL/L).
- Trace element solution: FeCl_3_ (50 mM), CaCl_2_ (20 mM), MnCl_2_ (10 mM), ZnSO_4_ (10 mM), CoCl_2_ (2 mM), CuCl_2_ (2 mM), NiCl_2_ (2 mM), Na_2_MoO_4_ (2 mM), Na_2_SeO_3_ (2 mM) and H_3_BO_3_ (2 mM).

### Biotransformation procedures

#### General procedure for screening experiments

The suitable strain was grown in a 1-L Erlenmeyer flask, sealed with a cotton plug, containing 300 mL of a nutrient-rich medium (PGM1 for *Pycnoporus cinnabarinus* CBS 353.63, *Lepista nuda*, *Agrocybe aegerita*, *Pleurotus sapidus*, *Pleurotus eryngii* and *Tricholoma terreum*; PGM2 for *P. cinnabarinus* DSM 3022, *P. cinnabarinus* DSM 15225, *Poria placenta*, *Inonotus radiatus*, *Armillaria mellea*, *Laetiporus sulphureus* and *Pholiota aurivella*). After complete growth, the biomass was collected by centrifugation, washed twice with some BM medium and added to a 1-L aerated flask containing 250 mL of fresh BM. Then, the suitable benzoic acid derivative was added as an aqueous solution of its ammonium salt (final acid concentration, 1.5 g/L). The flask was stirred at 130 rpm at 23 °C for 7 days. The biotransformation was monitored by daily sampling, followed by GC–MS and HPLC analysis.

#### General procedure for preparative biotransformations

The mycelium of the suitable strain was grown and collected as described above and was added to a 1-L aerated flask containing 250 mL of fresh BM and XAD1180N resin (5 g). The suitable benzoic acid derivative was added as an aqueous solution of its ammonium salt (final acid concentration, 1.5 g/L) and the flask was stirred at 130 rpm at 23 °C for 3–8 days. The biotransformation was worked up by filtration of the resin and fungal biomass through a sintered glass filter (porosity grade 0). The filtrate was extracted three times with diethyl ether (3 × 100 mL), and the organic phase was washed with brine, dried (Na_2_SO_4_) and evaporated under reduced pressure. The resulting oil was purified by bulb-to-bulb distillation and the distillate was characterized by GC–MS and HPLC analysis. The extracts obtained from vanillic acid biotransformation were purified using a puriFlash apparatus with methylene chloride to methanol 98:2 (v/v). The fractions collected in this way were verified using the GC method, and finally, only those containing the pure product were combined.

#### Biotransformation of anisic acid (**9**) with *P.eryngii*

After 3 days of biotransformation, the work-up procedure followed by bulb-to-bulb distillation afforded 190 mg of a pale yellow oil containing *p*-anisaldehyde (91%), *p*-anisic acid (4%) and unidentified compounds (5%). Acidification and extraction of the biotransformation broth afforded 80 mg of anisic acid. Yield of *p*-anisaldehyde vs transformed *p*-anisic acid is 64%, and absolute yield 51%.

#### Biotransformation of vanillic acid (**10**) with *P.cinnabarinus* DSM 3022

After 2 days of biotransformation, the work-up procedure followed by puriFlash separation afforded 178.3 mg of a pale yellow solid substance containing vanillin (95%) and vanillic acid (5%). Absolute yield is 30%.

#### Biotransformation of veratric acid (**11**) with *L.nuda*

After 8 days of biotransformation, the work-up procedure followed by bulb-to-bulb distillation afforded 210 mg of a yellow oil containing veratraldehyde (78%), veratryl alcohol (16%), veratric acid (4%) and unidentified compounds (2%). Acidification and extraction of the biotransformation broth afforded 120 mg of veratric acid. Yield of veratraldehyde vs transformed veratric acid is 73%, and absolute yield 48%.

#### Biotransformation of piperonylic acid (**12**) with* L. nuda*

After 4 days of biotransformation, the work-up procedure followed by bulb-to-bulb distillation afforded 170 mg of a pale yellow oil that solidified on standing, containing piperonal (89%), piperonyl alcohol (5%), piperonylic acid (4%) and unidentified compounds (2%). Acidification and extraction of the biotransformation broth afforded 145 mg of piperonylic acid. Yield of piperonal vs transformed piperonylic acid is 74%, and absolute yield 45%.

#### Biotransformation of 3,4,5-trimethoxybenzoic acid(**13**) with *P.*cinnabarinus CBS 353.63

After 4 days of biotransformation, the work-up procedure followed by bulb-to-bulb distillation afforded 256 mg of a pale yellow oil that solidified on standing, containing 3,4,5-trimethoxybenzaldehyde (83%) and 3,4,5-trimethoxybenzyl alcohol (17%). Acidification and extraction of the biotransformation broth afforded 35 mg of 3,4,5-trimethoxybenzaldehyde and 25 mg of 3,4,5-trimethoxybenzyl alcohol. Yield of 3,4,5-trimethoxybenzaldehyde is 71%.

### Instruments and analytic conditions

TLC: *Merck* silica gel 60 F_254_ plates. Column chromatography: silica gel.

Mass spectrum was recorded on a Bruker ESQUIRE 3000 PLUS spectrometer (ESI detector) or by GC–MS analyses.

GC–MS analyses: HP-6890 gas chromatograph equipped with a 5973 mass detector, using a HP-5MS column (30 m × 0.25 mm, 0.25 μm film thickness; Hewlett Packard) with the following temp. program: 38° (9 min)− 3°/min−90° (1 min) −6°/min−180° (1 min) −15°/min−280° (5 min); carrier gas, He; constant flow 1 mL/min; split ratio, 1/30; *t*_R_ given in minutes:

- Aldehydes: *t*_R_(**2**) 13.75, *t*_R_(**3**) 16.59, *t*_R_(**4**) 18.46, *t*_R_(**5**) 15.75 and *t*_R_(**6**) 20.90.
- Benzyl alcohols: *t*_R_(**14**) 13.92; *t*_R_(**15**) 17.86; *t*_R_(**16**) 19.03; *t*_R_(**17**) 16.82; *t*_R_(**18**) 21.66.
- Acid: *t*_R_(**10**) 20.34.

Benzoic acid methyl esters: *t*_R_(*p*-anisic acid methyl ester) 16.70; *t*_R_(veratric acid methyl ester) 20.37; *t*_R_(piperonylic acid methyl ester) 18.71; *t*_R_(eudesmic acid methyl ester) 22.49.

The mass spectra of *p*-anisaldehyde (**2**, Figure S1), vanillin (**3**, Figure S4), veratraldehyde (**4**, Figure S7), piperonal (**5**, Figure S10), 3,4,5-trimethoxybenzaldehyde (**6**, Figure S13), *p*-anisyl alcohol (**14**, Figure S2), vanillyl alcohol (**15**, Figure S5), veratryl alcohol (**16**, Figure S8), piperonyl alcohol (**17**, Figure S11), 3,4,5-trimethoxybenzyl alcohol (**18**, Figure S14), *p*-anisic acid methyl ester (Figure S3), vanillic acid (**10**, Figure S6), veratric acid methyl ester (Figure S9), piperonylic acid methyl ester (Figure S12) and eudesmic acid methyl ester (Figure S15) are reported in the Supplementary Information.

GC–MS analysis procedure: a sample of the biotransformation mixture (2 mL) was acidified by addition of diluted HCl (3% w/v) and then was extracted with dichloromethane (2 mL). The organic phase was separated, was treated with an excess of an ethereal solution of freshly prepared diazomethane and then was submitted to GC–MS analysis. Due to the partial methylation of the phenolic hydroxyl group, the diazomethane treatment was omitted for the biotransformation experiments involving vanillic acid (**10**).

HPLC analysis: a sample of the biotransformation mixture (2 mL) was acidified by addition of diluted HCl (3% w/v) and then was extracted with ethyl acetate (2 mL). The organic phase was separated, evaporated and suspended in the methanol (HPLC purity). Obtained samples were analysed using UltiMate 3000 (Dionex, Sunnyvale, CA, USA) with a UV detector and Luna 5u C18 column (25 cm × 4,6 mm, 5 μm; Phenomenex) by reverse-phase HPLC. The mobile phase consisted of aqueous 0.5% formic acid (solution A) and methanol (solution B) mixed A/B (v/v): 0 min (70:30), 11 min (25:75), 13 min (0:100) and 21 min (70:30) at rate 1 mL/min. The absorbance was measured at 254 nm, *t*_R_ given in minutes. Aldehydes: *t*_R_(**2**) 12.23, *t*_R_(**3**) 9.40, *t*_R_(**4**) 10.80, *t*_R_(**5**) 11.630 and *t*_R_(**6**) 11.80. Benzyl alcohols: *t*_R_(**14**) 10.42; *t*_R_(**15**) 5.72; *t*_R_(**16**) 8.91; *t*_R_(**17**) 10.207; *t*_R_(**18**) 9.66. Acids: *t*_R_(**9**) 12.40, *t*_R_(**10**) 8.53, *t*_R_(**11**) 10.70, *t*_R_(**12**) 12.083 and *t*_R_(**13**) 11.70*.*

## Results

In nature, the degradation of wood is accomplished primarily by fungi. Depending upon their mode of access to nutrient sources, Basidiomycetes are classified as saprophytic, parasitic and ectomycorrhizal species. In particular, saprophytic species are producers of a number of oxidoreductases and hydrolytic enzymes able to break down the lignocellulose biomass (Schmidt-Dannert 2016; Bilal et al. 2017). Since lignin is a complex phenylpropanoid polymer, the latter strains are able to transform the lignin monomers, at least to a certain extent. Therefore, it is not surprising that various saprophytic species have already been employed for the biotransformation of substituted benzoic acids. Among this class of microorganisms, ligninolytic species such as white-rot fungi and brown-rot fungi are the most investigated and promising.

According to this reasoning, we carefully chose several Basidiomycota strains, mainly composed of wood- or litter-degrading species, to be employed as whole-cell biocatalysts in our study. Due to the main application of the above-described aldehydes as a food additives, we selected species belonging to biosafety level 1, with a strong preference for those recognized as safe. The first selected species was *Pycnoporus cinnabarinus*, a well-known shelf-mushroom that has already been used for the reduction of vanillic acid (**10**) (Stentelaire et al. 2000). In order to investigate in deep the biocatalytic potential of this Basidiomycota, we employed three different strains belonging to the latter species. Hence, we selected further strains of white-rot fungi, such as two *Pleurotus* species, namely *P. sapidus* and *P. eryngii*, two *Armillaria mellea* strains and a single strain of *Pholiota aurivella*, *Agrocybe aegerita* and *Inonotus radiatus*. Moreover, we singled out some representative species of brown-rot fungi, namely two strains of *Poria placenta* and two strains of *Laetiporus sulphureus*.

All the above-described strains fulfil the safety requirements related to flavour production. In addition, they were selected among the Basidiomycota species, available from our collections, which have displayed reductive activity on benzoic acid derivatives. Finally, concerning fungi not classified as wood-decay species, we selected *Lepista nuda* and *Tricholoma terreum*, which are saprophytic and ectomycorrhizal species, respectively. Some preliminary experiments pointed to the potential of *Lepista nuda* in the reduction of carboxylic acids. Although this species does not degrade effectively lignocellulose, we decided to test its biocatalytic activity on the reduction of the selected benzoic acids. Ectomycorrhizal fungi are thought to not possess biocatalytic activity related to phenylpropanoid transformation. This is why we employed only one ectomycorrhizal strain, which was used as proof to confirm the above-mentioned general assumption.

From an experimental standpoint, all the selected strains were put in contact with the benzoic acids **9–13** (Fig. 3), and the biotransformation reactions were analysed at a regular periods of times, to spot the CAR activity of each species. We established a procedure based on using two media: a nutrient medium that allows mycelium growth and a biotransformation medium that can support the bioreduction of the benzoic acid derivatives to give the corresponding aldehydes. Indeed, ligninolytic fungi are producers of laccases and peroxidases (Singh Arora and Kumar Sharma 2010) that catalyse the oxidation and the polymerization of the oxy-functionalized benzoic acids. This biochemical activity and the reductase activity significantly increase when the mycelium is growing in a glucose-rich medium. Otherwise, a medium containing only oligo- or polysaccharides slows down the fungal metabolism, decreasing the side reactions and the alcohol dehydrogenase (ADH) activity, without relevant inhibition of the carboxylic acid reductase (CAR) activity (Stentelaire et al. 2000; Lesage-Meessen et al. 2002).

**Fig. 4:**
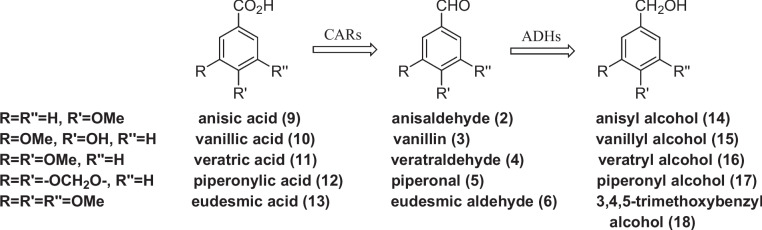
The basidiomycete-mediated reduction of the benzoic acid derivatives **9**–**13** can afford benzaldehydes **2–6** and benzyl alcohols **14–18**, thanks to the catalytic activity of carboxylic acid reductases (CARs) and alcohol dehydrogenases (ADHs), respectively

Our preliminary experiments showed that the main part of the tested strains, in glucose-rich medium, rapidly transformed the benzoic acids **9–13**, with simultaneous formation of polymeric materials and benzyl alcohol derivatives (**14–18**) (Fig. 4). However, the formation of substituted benzaldehydes was not observed. Thus, in our screening experiments, we adopted a general protocol that provides for fungal growth in a glucose-rich medium followed by mycelium filtration and the replacement of the growth medium with a biotransformation medium containing only the disaccharide maltose (20 g/L) as a carbohydrate source. Then, the ammonium salts of the suitable benzoic acid derivatives **9–13** were added at once, and the biotransformation experiments were performed aerobically under vigorous shaking. The progress of the reactions was evaluated by GC–MS or HPLC analysis. This analytic technique allows measuring the relative amount of all the volatile products formed by the biotransformation of the starting benzoic acids.

The results of our screening are collected in Table 1, 2, 3, 4 and 5, whose comprehensive discussion is reported in the next paragraph.

**Table 1:** Biotransformation of *p*-anisic acid **9** with the selected Basidiomycota strains

Entry	Strain	Time (days)	Acid (%)^1^	Aldehyde (%)^1^	Benzylic alcohol (%)^1^	Others^1,2^
1	*P. cinnabarinus* CBS 353.63	1	8	23	68	1
		3	5	15	80	-
		5	0.5	9	90	0.5
2	*P. cinnabarinus* DSM 3022	1	100	-	-	-
		3	92	2	6	-
		5	92	4	4	-
3	*P. cinnabarinus* DSM 15225	1	45	44	11	-
		2	-	33	67	-
		3	-	9	91	-
4	*P. sapidus* DSM 8266	1	3	97	-	-
		3	1	98	-	< 1
		7	23	22	-	55
5	*P. eryngii* DSM 114648	1	8	92	-	-
		2	2.5	97.5	-	-
		4	0.2	99.8	-	-
6	*A. mellea* AM296	3	100	-	-	-
		7	-	-	-	-
7	*A. mellea* AM461	7	100	-	-	-
8	*P. aurivella* AM 522	3	-	-	-	-
9	*A. aegerita* DSM 22459	3	100	-	-	-
		7	-	5	95	-
10	*I. radiatus* AM 70	3	34	3	63	-
		7	-	3	97	-
11	*P. placenta* AM36	3	80	-	-	20
		7	-	-	-	-
12	*P. placenta* AM38	7	100	-	-	-
13	*L. sulphureus* AM498	7	100	-	-	-
14	*L. sulphureus* AM515	7	100	-	-	-
15	*L. nuda* DSM 115118	1	68	25	2	5
		3	16	59	20	5
		7	2	54	31	13
16	*T. terreum* CBS 100138	7	100	-	-	-

^1^The reported data describes the composition of the biotransformation mixture measured through GC–MS analysis. ^2^This data indicates the overall percentage of all compounds, different from acid, aldehyde and benzylic alcohol that were detected by the GC–MS analysis

**Table 2:** Biotransformation of veratric acid **11** with the selected Basidiomycota strains

Entry	Strain	Time (days)	Acid (%)^1^	Aldehyde (%)^1^	Benzylic alcohol (%)^1^	Others^1,2^
1	*P. cinnabarinus* CBS 353.63	2	85	14	-	1
		5	50	47	2	1
		10	-	82	17	1
2	*P. cinnabarinus* DSM 3022	1	2	17	81	-
		3	-	3	95	2
3	*P. cinnabarinus* DSM 15225	1	100	-	-	-
		3	16	69	15	-
		5	-	62	38	-
4	*P. sapidus* DSM 8266	1	82	17	-	1
		4	29	71	-	-
		7	-	88	-	12
5	*P. eryngii* DSM 114648	7	100	-	-	-
6	*A. mellea* AM296	3	100	-	-	-
		7	-	-	-	-
7	*A. mellea* AM461	3	-	-	-	-
8	*P. aurivella* AM 522	3	95	-	-	5
		7	90	-	-	10
9	*A. aegerita* DSM 22459	3	-	-	-	-
10	*I. radiatus* AM 70	3	80	9	11	-
		7	60	10	30	-
		14	35	-	65	-
11	*P. placenta* AM36	7	-	-	-	-
12	*P. placenta* AM38	7	100	-	-	-
13	*L. sulphureus* AM498	3	10	55	35	-
		7	-	15	85	
14	*L. sulphureus* AM515	7	100	-	-	-
15	*L. nuda* DSM 115118	3	84	15	-	1
		7	3	90	7	-
16	*T. terreum* CBS 100138	7	100	-	-	-

^1^The reported data describes the composition of the biotransformation mixture measured through GC–MS analysis. ^2^This data indicates the overall percentage of all compounds, different from acid, aldehyde and benzylic alcohol that were detected by the GC–MS analysis

**Table 3:** Biotransformation of eudesmic acid **13** with the selected Basidiomycota strains

Entry	Strain	Time (days)	Acid (%)^1^	Aldehyde (%)^1^	Benzylic alcohol (%)^1^	Others^1,2^
1	*P. cinnabarinus* CBS 353.63	1	63	25	12	-
		3	2	60	38	-
		7	-	-	100	-
2	*P. cinnabarinus* DSM 3022	1	57	14	29	-
		3	-	3	97	2
3	*P. cinnabarinus* DSM 15225	1	94	3	3	-
		3	33	51	16	-
		5	-	37	63	-
4	*P. sapidus* DSM 8266	1	100	-	-	-
		4	-	22	78	-
		7	-	10	90	-
5	*P. eryngii* DSM 114648	7	100	-	-	-
6	*A. mellea* AM296	3	100	-	-	-
		7	-	-	-	-
7	*A. mellea* AM461	7	100	-	-	-
8	*P. aurivella* AM 522	3	< 1	3	97	-
		7	-	3	97	-
9	*A. aegerita* DSM 22459	3	100	-	-	-
10	*I. radiatus* AM 70	3	95	-	5	-
		7	85	-	15	-
		14	45	-	55	-
11	*P. placenta* AM36	3	< 1	-	-	-
12	*P. placenta* AM38	3	60	-	35	5
		7	-	-	90	10
13	*L. sulphureus* AM498	3	100	-	-	-
		7	80	-	-	20
14	*L. sulphureus* AM515	7	100	-	-	-
15	*L. nuda* DSM 115118	7	100	-	-	-
16	*T. terreum* CBS 100138	7	100	-	-	-

^1^The reported data describes the composition of the biotransformation mixture measured through GC–MS analysis. ^2^This data indicates the overall percentage of all compounds, different from acid, aldehyde and benzylic alcohol that were detected by the GC–MS analysis

**Table 4:** Biotransformation of vanillic acid **10** with the selected Basidiomycota strains

Entry	Strain	Time (days)	Acid (%)^1^	Aldehyde (%)^1^	Benzylic alcohol (%)^1^	Others^1,2^
1	*P. cinnabarinus* CBS 353.63	3	100	-	-	-
		7	-	-	-	-
2	*P. cinnabarinus* DSM 3022	1	-	100	-	-
		2	-	100	-	-
3	*P. cinnabarinus* DSM 15225	1	-	-	-	-
4	*P. sapidus* DSM 8266	3	-	-	-	-
5	*P. eryngii* DSM 114648	7	100	-	-	-
6	*A. mellea* AM296	3	100	-	-	-
		7	-	-	-	-
7	*A. mellea* AM461	3	100	-	-	-
		7	-	-	-	-
8	*P. aurivella* AM 522	3	-	-	-	-
9	*A. aegerita* DSM 22459	3	-	-	-	-
10	*I. radiatus* AM 70	3	60	17	23	-
		7	-	-	100	-
		14	-	-	-	-
11	*P. placenta* AM36	3	100	-	-	-
		7	-	-	-	-
12	*P. placenta* AM38	3	-	-	-	100
13	*L. sulphureus* AM498	3	-	-	-	100
14	*L. sulphureus* AM515	3	-	-	20	80
		7	-	-	-	100
15	*L. nuda* DSM 115118	3	-	-	-	-

^1^The reported data describes the composition of the biotransformation mixture measured through GC–MS analysis. ^2^This data indicates the overall percentage of all compounds, different from acid, aldehyde and benzylic alcohol that were detected by the GC–MS analysis

**Table 5:** Biotransformation of piperonylic acid **12** with the selected Basidiomycota strains

Entry	Strain	Time (days)	Acid (%)^1^	Aldehyde (%)^1^	Benzylic alcohol (%)^1^	Others^1,2^
1	*P. cinnabarinus* CBS 353.63	1	39	15	46	-
		3	76	-	24	-
2	*P. cinnabarinus* DSM 3022	3	100	-	-	-
		5	100	-	-	-
3	*P. cinnabarinus* DSM 15225	1	77	12	3	8
		3	76	11	5	8
		5	80	7	8	5
4	*P. sapidus* DSM 8266	1	64	36	-	-
		2	56	44	-	-
		4	62	38	-	-
		7	76	9	-	15
5	*P. eryngii* DSM 114648	1	78	16	-	6
		3	83	17	-	-
		10	100	-	-	-
6	*P. aurivella* AM 522	7	100	-	-	-
7	*A. aegerita* DSM 22459	7	100	-	-	-
8	*I. radiatus* AM 70	7	100	-	-	-
9	*P. placenta* AM36	4	90	3	7	-
		7	90	3	7	-
10	*L. sulphureus* AM498	4	90	5	5	-
		7	-	-	-	-
11	*L. sulphureus* AM515	4	95	3	2	-
		7	46	24	32	-
12	*L. nuda* DSM 115118	1	40	48	12	-
		3	-	44	56	-

^1^The reported data describes the composition of the biotransformation mixture measured through GC–MS analysis. ^2^This data indicates the overall percentage of all compounds, different from acid, aldehyde and benzylic alcohol that were detected by the GC–MS analysis

The next step of our experimental work was the devising of a reliable preparative process. Since high concentrations of the produced benzaldehydes are toxic to the fungal strains, which reduced them to the corresponding benzylic alcohols, we decided to perform the biotransformations in the presence of the polystyrene resin Amberlite® XAD1180N. The latter polymer is commercialized as small beads, which are completely insoluble in water and possess a very high chemical stability. This non-polar material, once suspended in the fermentation broth, adsorbs efficiently all the neutral organic compounds formed during biotransformation. Differently, organic salts or very polar compounds are not adsorbed and remain in aqueous phase. In the presence of Amberlite, the substituted benzaldehydes were adsorbed efficiently as soon as they were formed whereas the substrates, namely the ammonium salts of the suitable benzoic acids, remained mainly in the fermentation broth. Low concentration of benzaldehydes in the biotransformation medium helps to reduce the formation of the benzyl alcohols and of dimeric or polymeric side products. In addition, the work-up procedure was greatly simplified. Resin and fungal hyphae were collected by simple filtration and the following extraction of the filtrate with ethyl acetate allowed the isolation of the crude biotransformation mixture, which can be purified by distillation and/or chromatography. The benzaldehydes are the main components of the distillate, eventually containing a minor amount of benzylic alcohols and unreacted benzoic acids.

The application of this protocol to the reduction of *p*-anisic (**9**), vanillic (**10**), veratric (**11**), piperonylic (**12**) and eudesmic (**13**) acids allowed obtaining, after a rough purification of the crude biotransformation mixtures, a product mixture containing anisaldehyde (**2**) (91%), vanillin (**3**) (95%), veratraldehyde (**4**) (78%), piperonal (**5**) (89%) and 3,4,5-trimethoxybenzaldehyde (**6**) (83%).

## Discussion

The biotransformation experiments performed using *p*-anisic acid (**9**) (Table 1) indicated that different strains possess CAR activity. Anisaldehyde (**2**) was produced by *P. cinnabarinus*, *P. sapidus*, *P. eryngii*, *A. aegerita*, *I. radiatus* and *L. nuda*.

Despite these good results, anisaldehyde (**2**) was further reduced producing a considerable amount of *p*-anisyl alcohol (**14**) and other unidentified products (entries 1, 3, 9, 10 and 15). Interestingly, the best results were obtained using the two *Pleurotus* species (entries 4 and 5) with the remarkable difference that *P. sapidus*, after long contact time, afford a significant amount of unidentified products, which were not formed by *P. eryngii*. The latter strain reduced almost completely *p*-anisic acid (**9**) into *p*-anisaldehyde (**2**) and turned out to be the most suitable microorganism to perform this transformation.

The modification of the chemical structure of the starting benzoic acid derivatives greatly affected the biotransformation results. More specifically, CAR enzymes produced by *P. eryngii* and *P. placenta* AM36 showed no activity against the derivatives containing one additional methoxy group (veratric acid **11**, Table 2) or two additional methoxy groups (eudesmic acid **13**, Table 3). Differently, *L. sulphureus* AM498, *P. aurivella* and *P. placenta* AM38, which were inactive with anisic acid (**9**), were able to reduce veratric acid (**11**) (Table 2, entry 13) or eudesmic acid (**13**) (Table 3, entries 8 and 12).

Concerning veratric acid (**11**), the three *P. cinnabarinus* strains, *P. sapidus*, *I. radiatus*, *L. sulphureus* AM515 and *L. nuda* reduced efficiently the substrate. Despite this fact, only *P. cinnabarinus* CBS 353.63, *P. sapidus* and *L. nuda* produced veratraldehyde (**4**) in high amount. In particular, *L. nuda* gave the best performance affording after 7 days of biotransformation a mixture of reduced products mainly consisting of veratraldehyde (**4**) (90%, Table 2, entry 15) together with a minor amount of the starting acid and veratryl alcohol (**16**).

Eudesmic acid (**13**) was efficiently reduced by the three *P. cinnabarinus* strains, *P. sapidus*, *P. aurivella*, *I. radiatus* and *P. placenta* AM38 (Table 3, entries 1–4, 8, 10 and 12) but all these strains produced relatively high amounts of the corresponding benzylic alcohol, which increased with longer contact time. Only *P. cinnabarinus* CBS 353.63, after 3 days of biotransformation, generated a significant amount of 3,4,5-trimethoxybenzaldehyde (**6**) (60%).

A further important observation regards *Tricholoma terreum* (entries 16 in Table 1, 2 and 3). None of the substrates was affected by *T. terreum*, clearly indicating that this ectomycorrhizal microorganism is completely inactive. It is worth noting that usually, methoxy-substituted benzoic acids are less toxic than the corresponding phenol-substituted and methylenedioxy-substituted derivatives. According to the latter considerations, we decided to exclude the latter strain from the experiments involving vanillic (**10**) and piperonylic (**12**) acids.

For the biotransformations involving the latter two acids (Table 4 and 5), we can observe a remarkable decrease of CAR activity, especially in the biotransformation of vanillic acid (**10**). All the evaluated strains transformed readily and completely the latter substrate **10**, with the single exception of *P. eryngii* which was inactive (Table 4, entry 5).

Despite the high reactivity observed, only *P. cinnabarinus* DSM 3022, *I. radiatus* and *L. sulphureus* AM515 (Table 4, entries 2, 10 and 14, respectively) were able to produce vanillin (**3**) and/or vanillyl alcohol (**15**). *I. radiatus* and *L. sulphureus* AM515 reduced vanillic acid (**10**) to the corresponding aldehyde with rather modest yields as the biotransformation reactions led to the production of different metabolites with complete transformation of the starting acids. Nevertheless, prolonging the biotransformation time, even these strains degraded completely the substrate and its derivatives. Surprisingly, out of three *P. cinnabarinus* strains, only DSM 3022 accumulated vanillin and the biotransformations performed with this microorganism proceeded with very high selectivity, without the formation of vanillyl alcohol. This result is remarkable because a prospective industrial process, based on this kind of biotransformation, could afford natural vanillin without any demanding purification procedure.

Interestingly, we underline the relevant discrepancy between the results of our experiments of vanillic acid reduction, performed with *P. cinnabarinus* CBS 353.63 and DSM 15225, and those described in the literature (Stentelaire et al. 2000; Lesage-Meessen et al. 2002). The latter authors efficiently reduced vanillic acid to vanillin using the *P. cinnabarinus* strain MUCL 39533, which is described as a laccase-deficient strain. Thus, we can suppose that the genetic differences among *P. cinnabarinus* strains, in terms of laccases expression, could justify the different results here presented. Accordingly, we presume that the latter enzymes are responsible for the rapid oxidation of vanillic acid, vanillin and vanillyl alcohol, thus leading to the complete elimination of both the substrate and the products deriving from its reduction. Phenol derivatives are the natural substrates of the laccases, which catalyse the formation of polymeric products. Therefore, the polymerization reaction is less relevant when the substrate does not contain free phenol groups. This aspect is confirmed by the biotransformation experiments that use benzoic acids **9** and **11–13** as substrates, where the substituents are only methoxy or methylenedioxy functional groups. In these cases, the substituted benzaldehydes and benzylic alcohols, deriving from the reduction reaction, can be accumulated in the fermentation medium easier than those obtained from vanillic acid.

Furthermore, we can observe that the biotransformation of veratric acid (**11**) and piperonylic acid (**12**), which are 3,4-dimethoxy and 3,4-methylenedioxy substituted, gave similar results (Table 2 and 5).

Piperonylic acid (**12**) is transformed by *P. cinnabarinus P. sapidus*, *P. eryngii*, *P. placenta*, *L. sulphureus* and *L. nuda*. As described for veratric acid (**11**), only *P. sapidus* and *L. nuda* are able to accumulate relevant amounts of the substituted benzaldehydes. The latter strain did not afford detectable amount of side products, which proved to be the most suitable microorganism for producing of both veratraldehyde (**4**) and piperonal (**5**).

It is worth noting that all the screening experiments described before have been conceived to assess the biocatalytic potentialities of the evaluated strains, regardless of their biotransformation efficiency. Overall, we established that *P. eryngii* and *P. cinnabarinus* (DSM 3022) are the most appropriate fungal strains for the production of *p*-anisaldehyde (**2**) and vanillin (**3**), respectively. *L. nuda* is the most efficient Basidiomycota for the synthesis of veratraldehyde (**4**) and piperonal (**5**) whereas *P. cinnabarinus* (CBS 353.63) is the only microorganism able to produce a significant amount of 3,4,5-trimethoxybenzaldehyde (**6**) by reduction of the corresponding acid.

In summary, our study demonstrates that different Basidiomycota strains can be exploited as whole-cell biocatalysts for the preparation of anisaldehyde, vanillin, veratraldehyde, piperonal and 3,4,5-trimethoxybenzaldehyde by reduction of the corresponding benzoic acid derivatives.

Starting from benzoic acids of natural origin, the obtained aldehydes can be commercialized as high-value natural flavours, in compliance with the European and USA regulation of food flavouring substances. This aspect is particularly relevant for aldehydes such as natural piperonal, which is characterized by very high commercial value and limited availability on the market.

In addition, our findings demonstrated the importance of strain variability and of biotransformation conditions to achieve a reliable process for substituted benzaldehydes production. The reported experiments showed that different strains belonging to the same fungal species can perform the investigated biotransformation in a different manner.

Finally, we underline the remarkable bioactivity of the Basidiomycota *Lepista nuda*. Interestingly, the latter saprophytic strain is not a wood-decay species but is able to transform some benzoic acid derivatives with a biocatalytic activity similar to that of the better-known and already investigated white-rot and brown-rot fungi.

## Supplementary Information

Below is the link to the electronic supplementary material.

Supplementary file1 (PDF 1242 KB)

## Data Availability

Data generated during this study are included in this published article and in its supplementary material file.
