# Supplementary material for: Basidiomycota strains as whole-cell biocatalysts for the synthesis of high-value natural benzaldehydes
Source: Appl Microbiol Biotechnol. 2024 Jan 11;108(1):113. doi: 10.1007/s00253-023-12872-y (PMC10784365; doi:10.1007/s00253-023-12872-y)

Supporting Information

Basidiomycota strains as whole-cell biocatalysts for the synthesis of high-value natural benzaldehydes

Stefano Serra[1,*], Stefano Marzorati[1], Ewa Szczepańska[2], Tomasz Strzała[3], Filip Boratyński[2,*],

1 Consiglio Nazionale delle Ricerche (C.N.R.), Istituto di Scienze e Tecnologie Chimiche, Via Mancinelli 7, 20131 Milano, Italy

2 Department of Food Chemistry and Biocatalysis, Wrocław University of Environmental and Life Sciences, Norwida 25, 50-375 Wrocław, Poland

3 Department of Genetics, Wroclaw University of Environmental and Life Sciences, ul. Kozuchowska 7, 51-631 Wrocław, Poland

Table of contents:

1. Figure S1: MS spectrum of *p*-anisaldehyde
2. Figure S2: MS spectrum of *p*-anisyl alcohol
3. Figure S3: MS spectrum of *p*-anisic acid methyl ester
4. Figure S4: MS spectrum of vanillin
5. Figure S5: MS spectrum of vanillyl alcohol
6. Figure S6: MS spectrum of vanillic acid
7. Figure S7: MS spectrum of veratraldehyde
8. Figure S8: MS spectrum of veratryl alcohol
9. Figure S9: MS spectrum of veratric acid methyl ester
10. Figure S10: MS spectrum of piperonal
11. Figure S11: MS spectrum of piperonyl alcohol
12. Figure S12: MS spectrum of piperonylic acid methyl ester
13. Figure S13: MS spectrum of 3,4,5-trimethoxybenzaldehyde
14. Figure S14: MS spectrum of 3,4,5-trimethoxybenzyl alcohol
15. Figure S15: MS spectrum of eudesmic acid methyl ester

Figure S1: MS spectrum of *p*-anisaldehyde

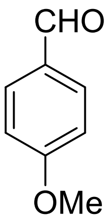

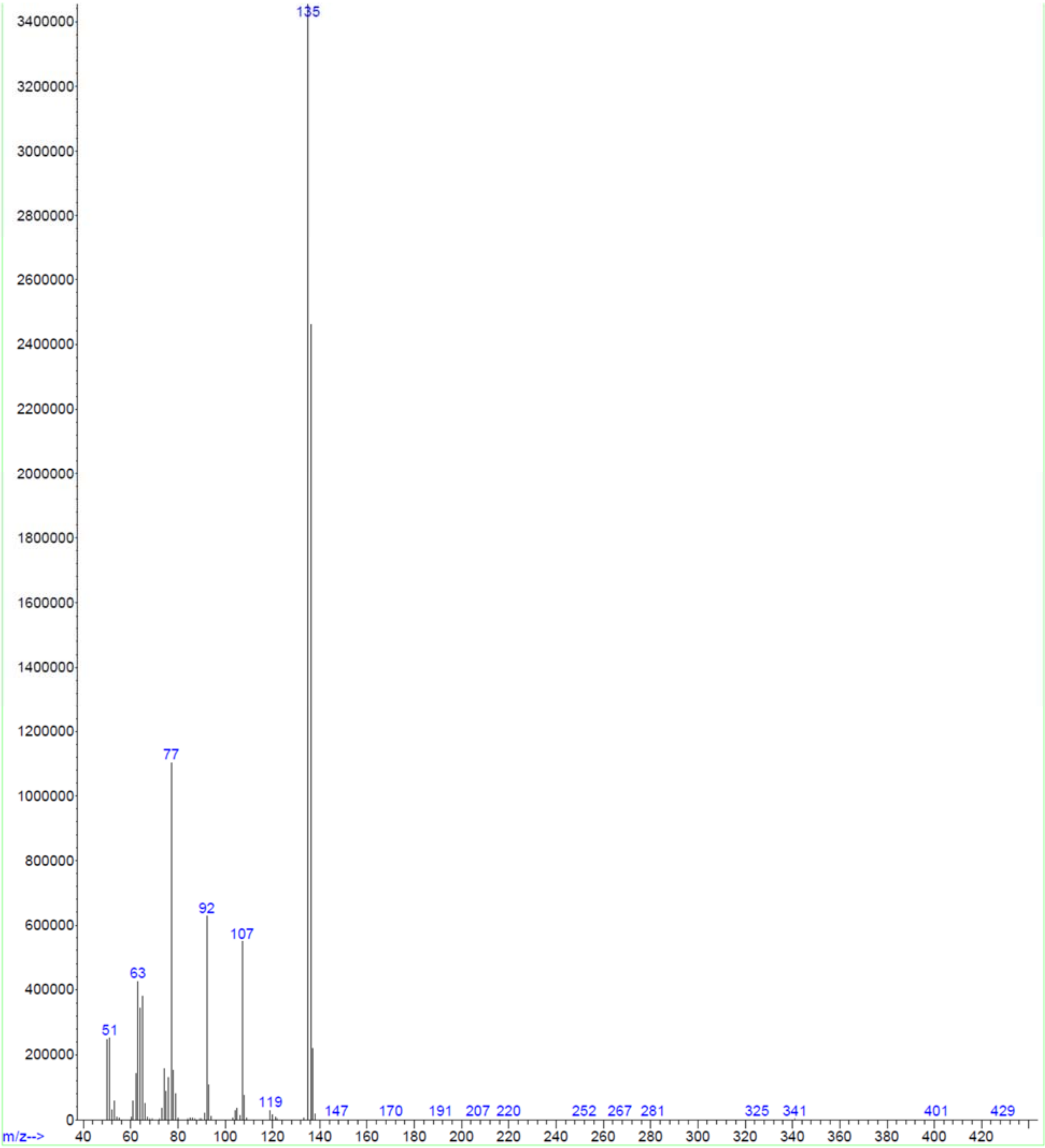

Figure S2: MS spectrum of *p*-anisyl alcohol

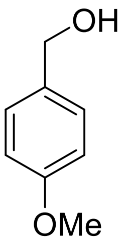

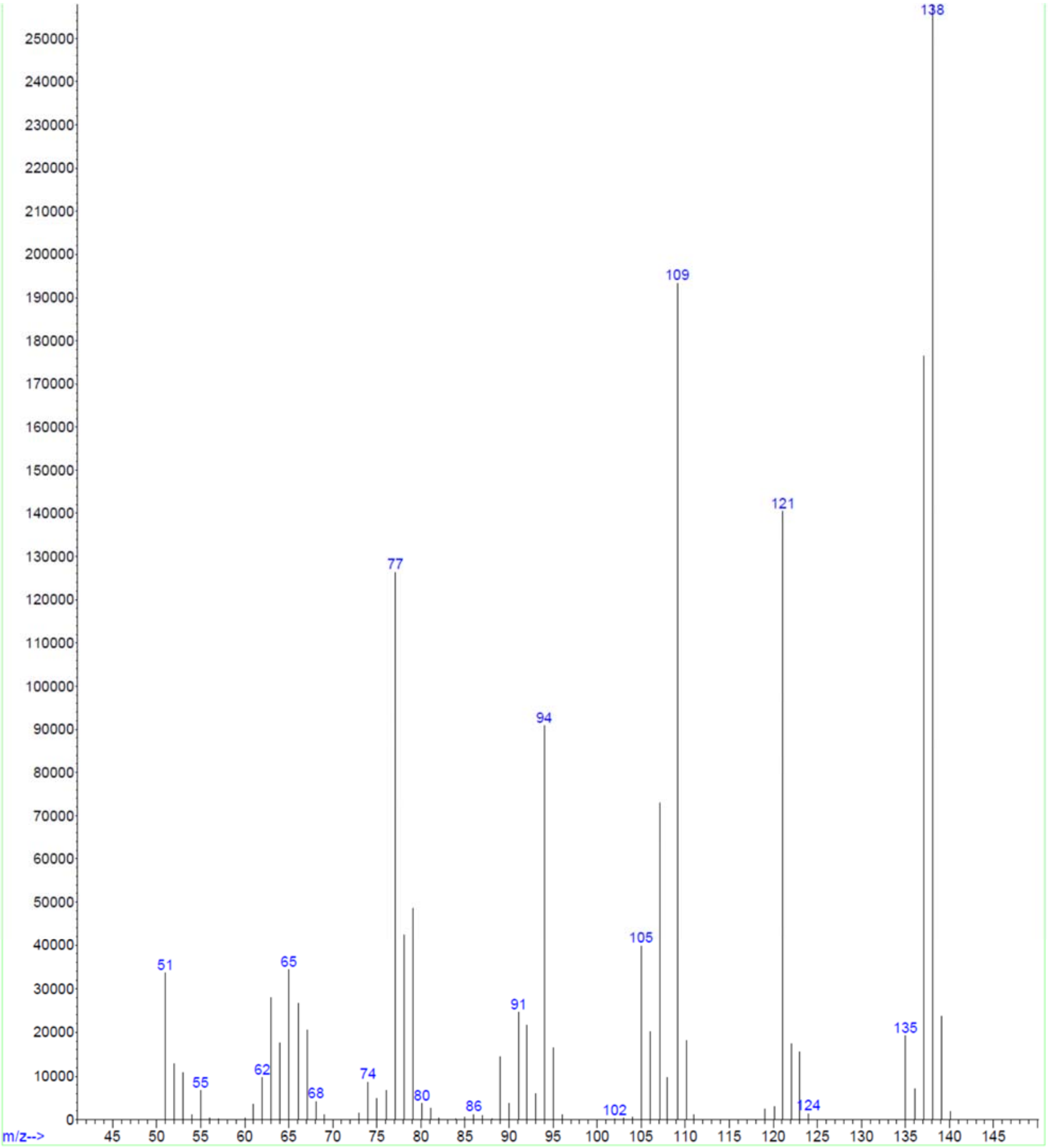

Figure S3: MS spectrum of *p*-anisic acid methyl ester

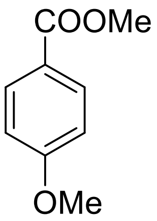

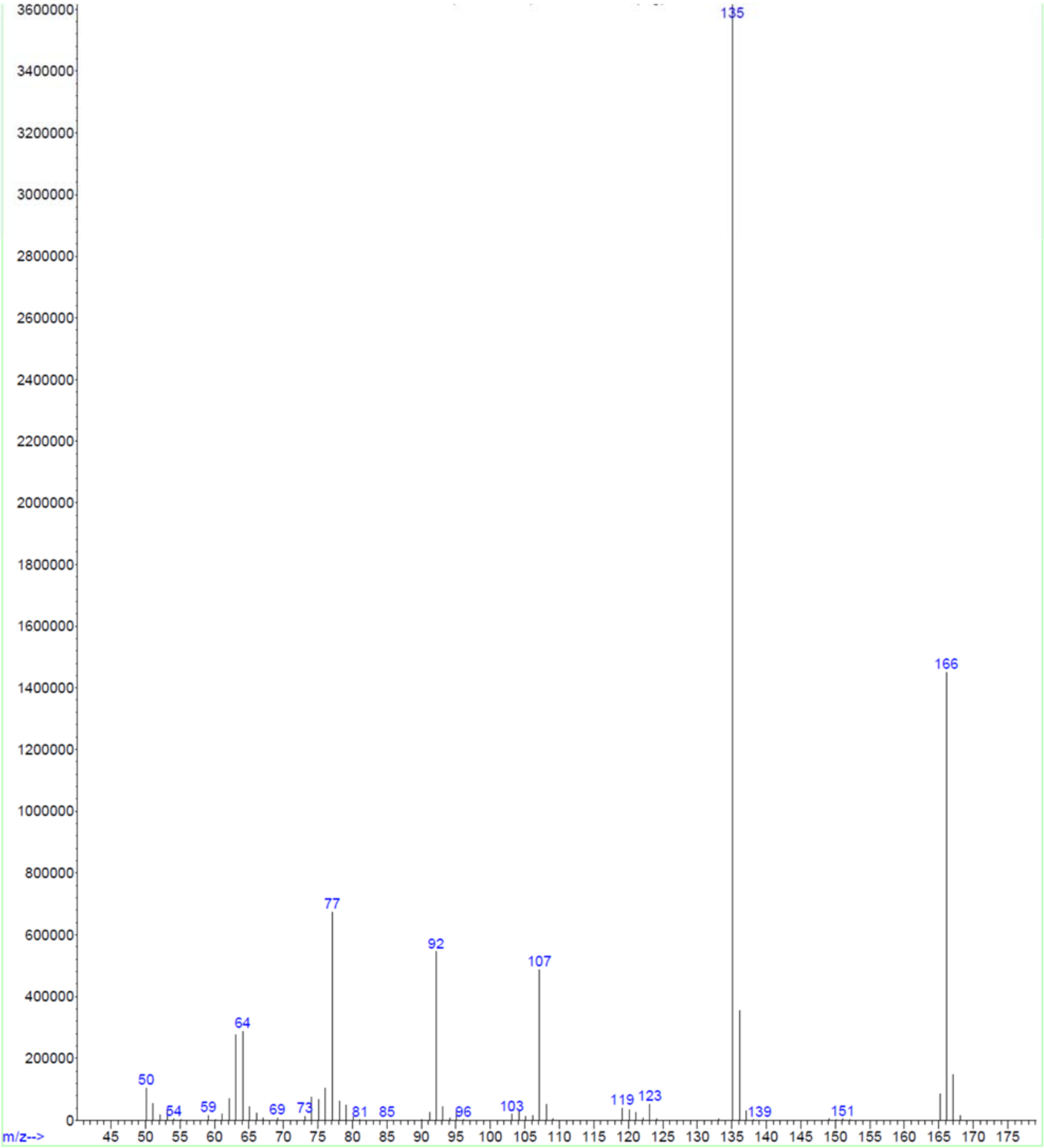

Figure S4: MS spectrum of vanillin

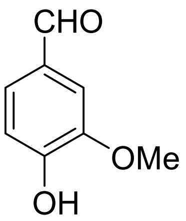

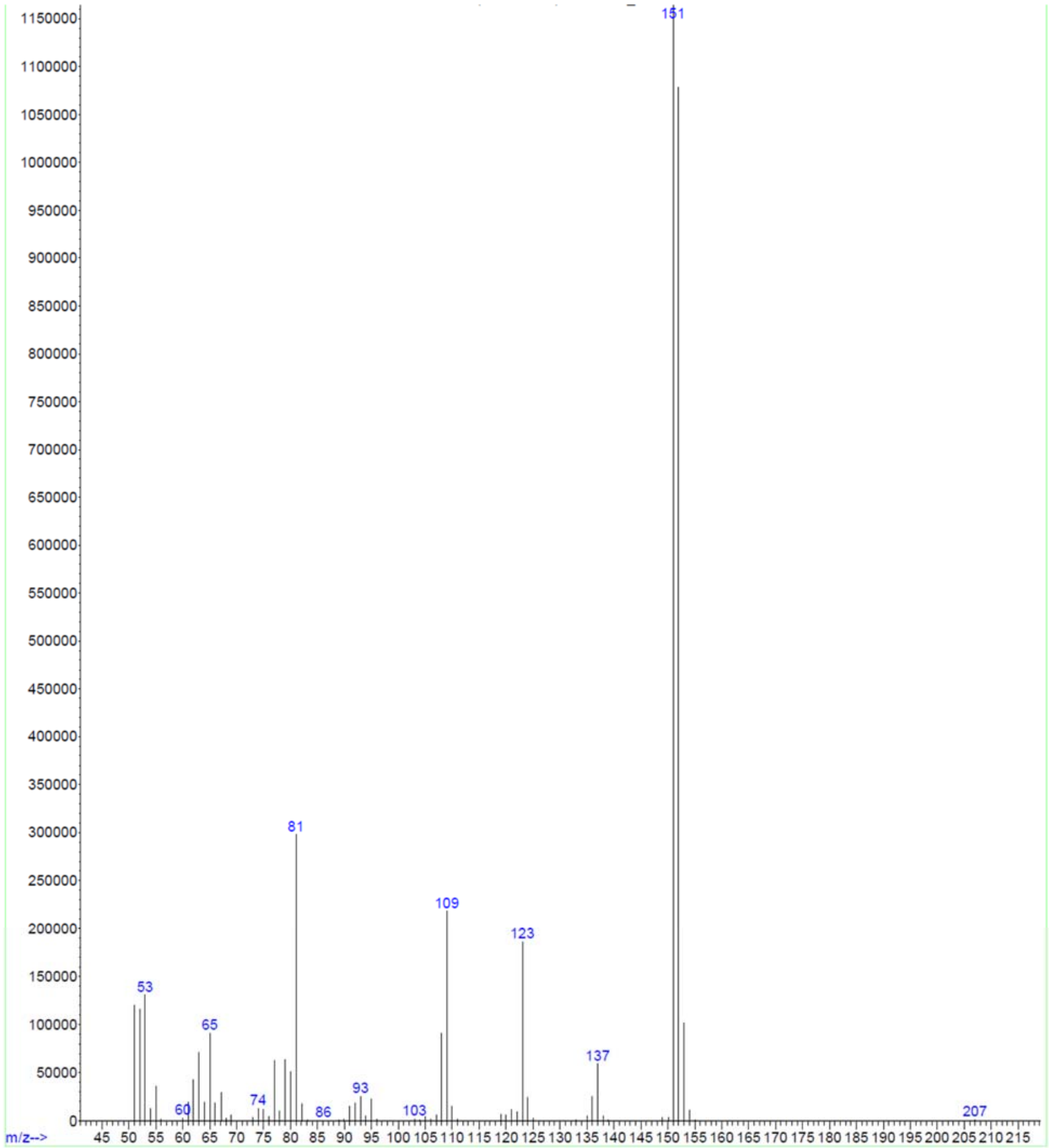

Figure S5: MS spectrum of vanillyl alcohol

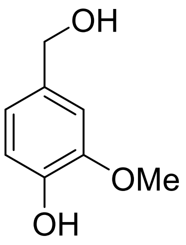

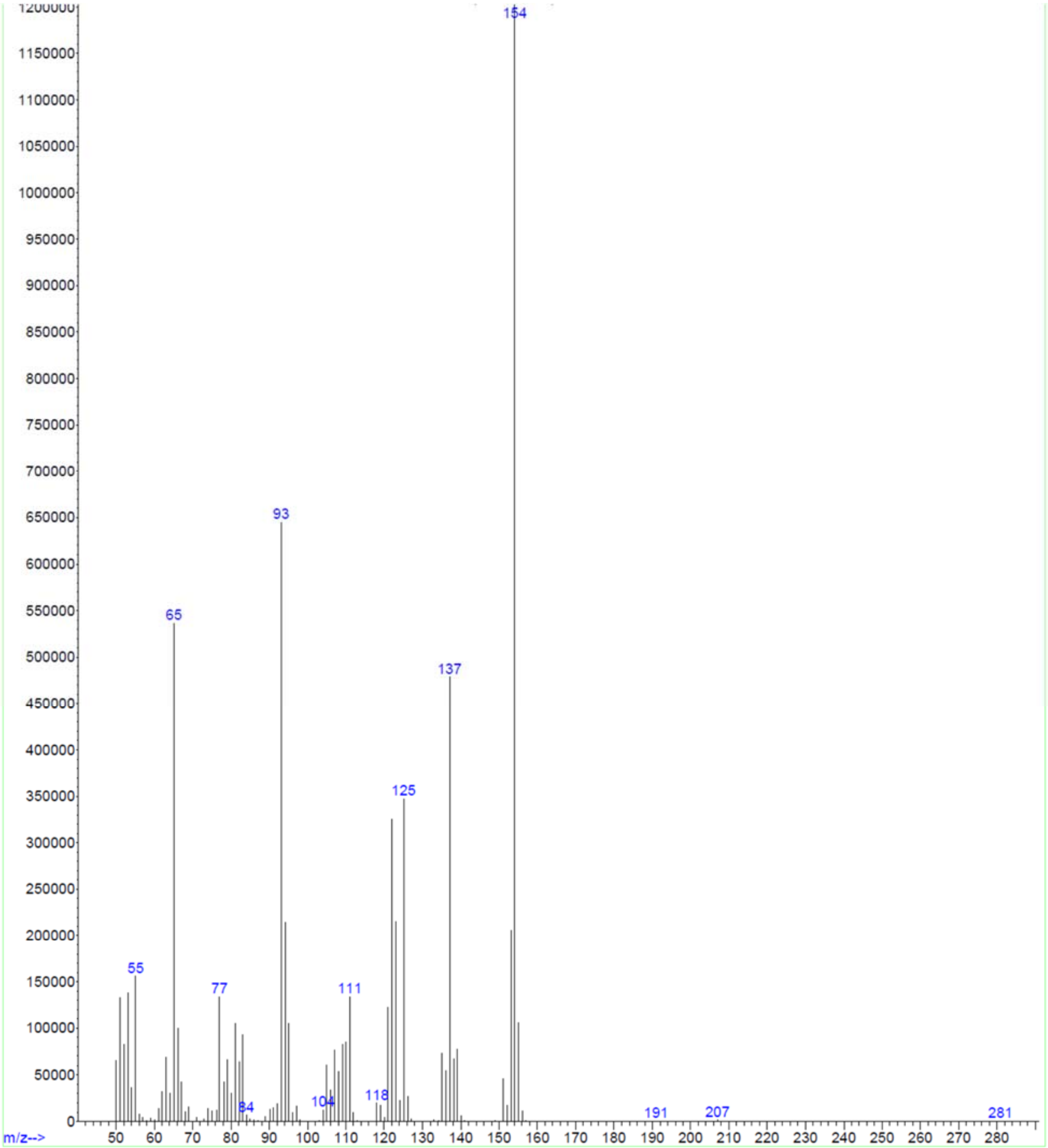

Figure S6: MS spectrum of vanillic acid

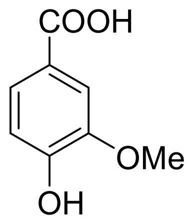

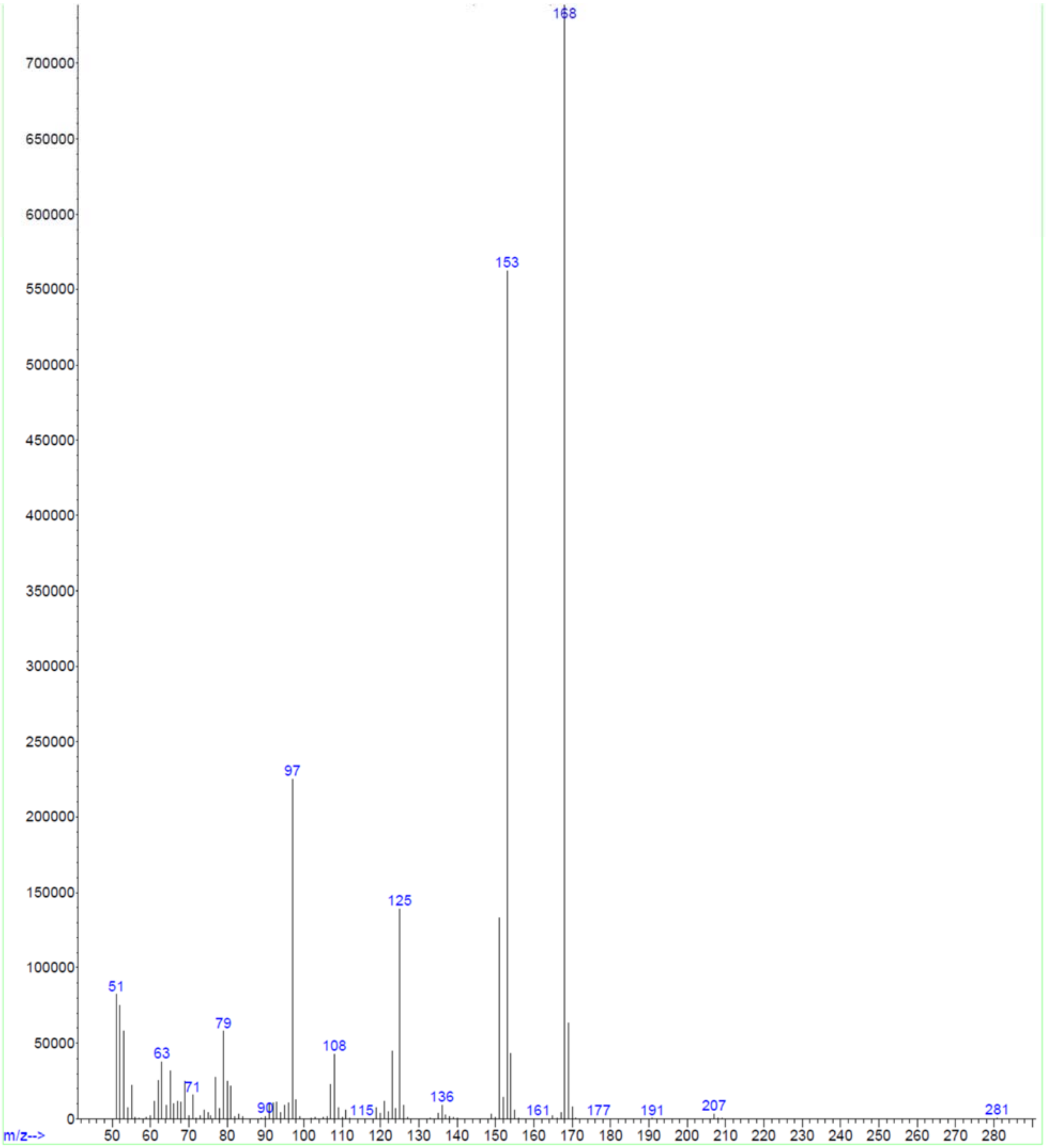

Figure S7: MS spectrum of veratraldehyde

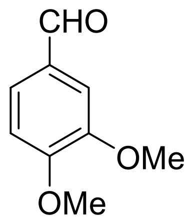

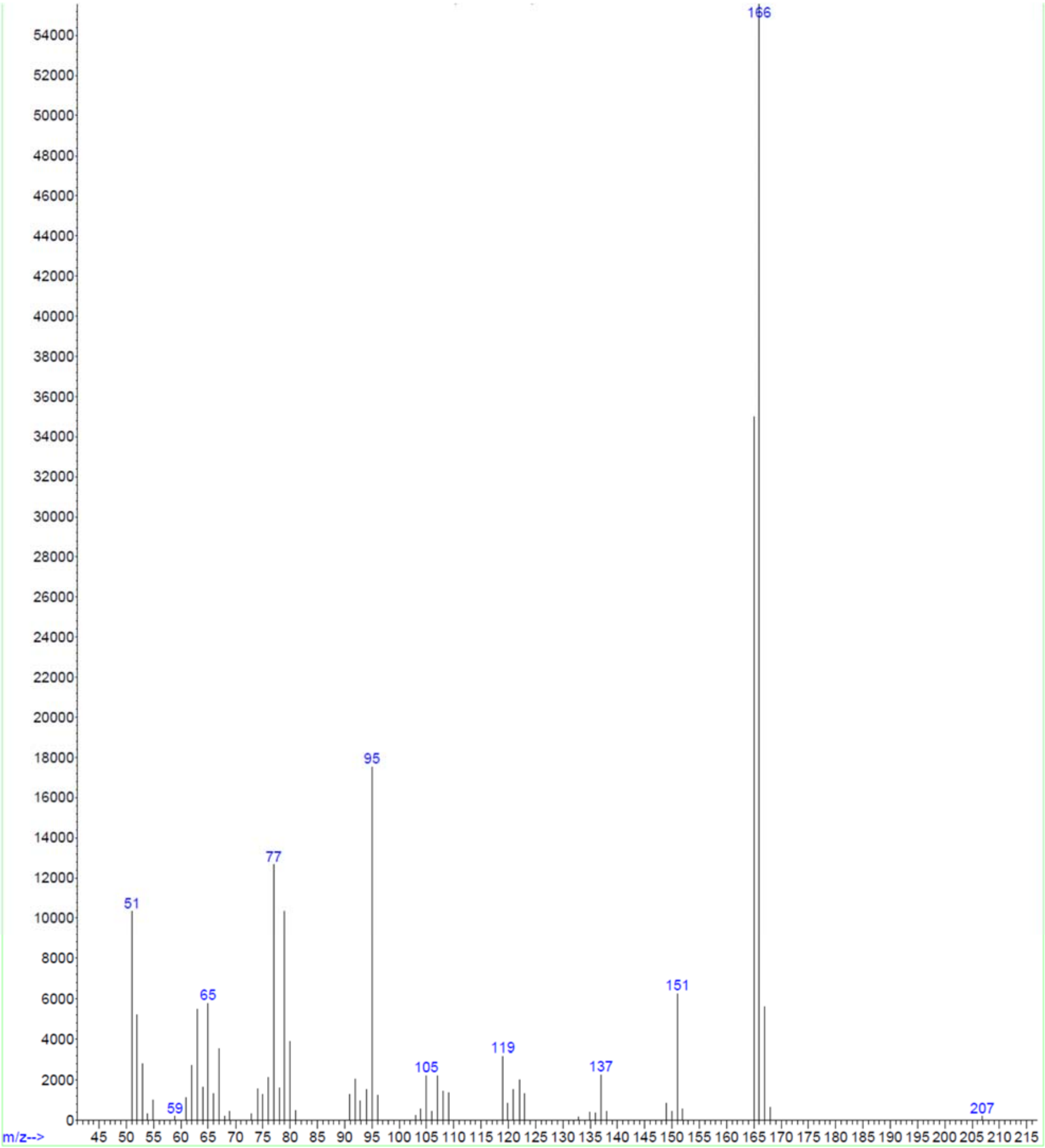

Figure S8: MS spectrum of veratryl alcohol

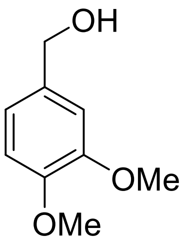

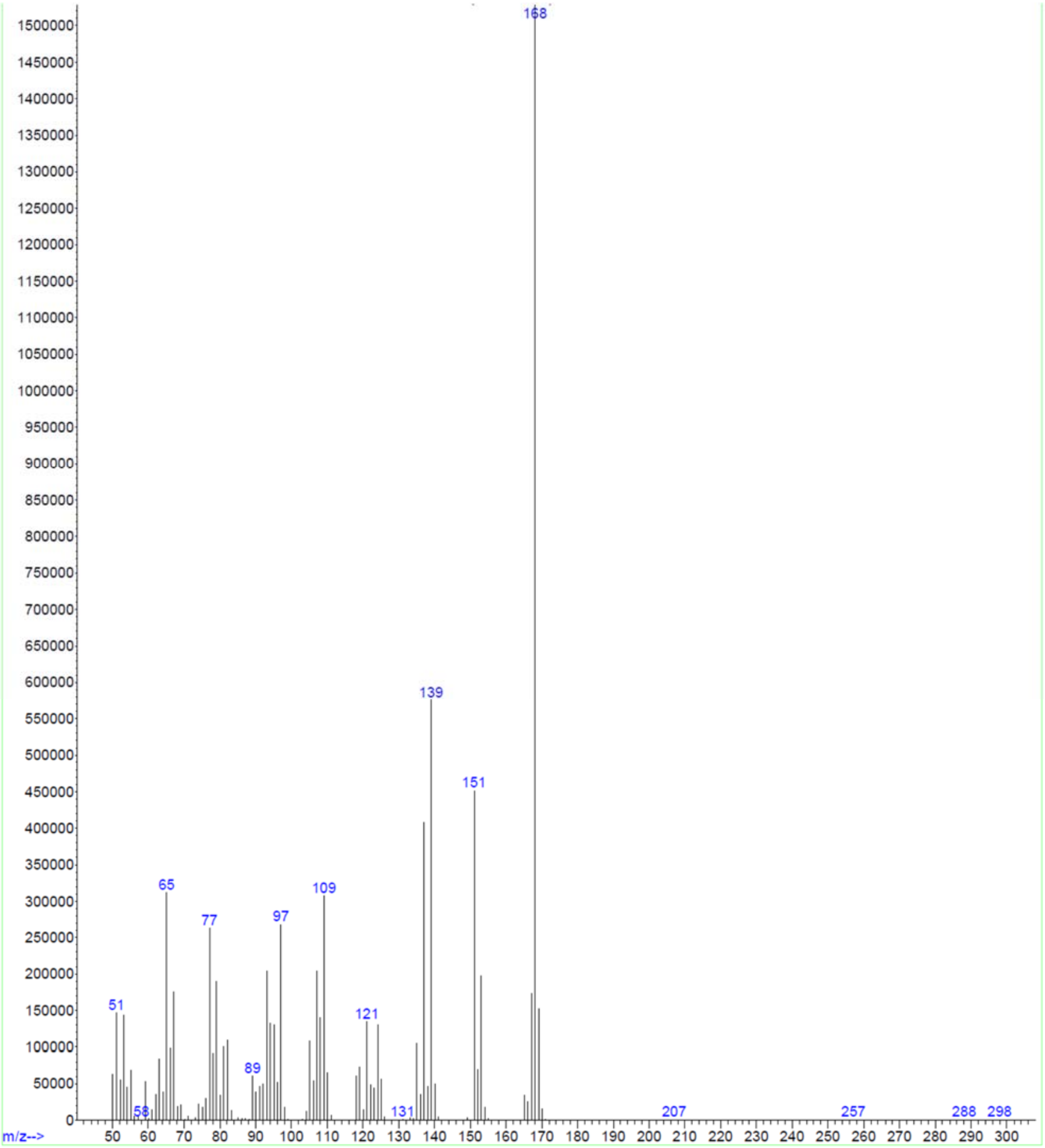

Figure S9: MS spectrum of veratric acid methyl ester

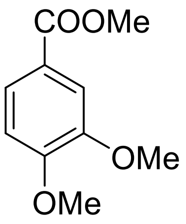

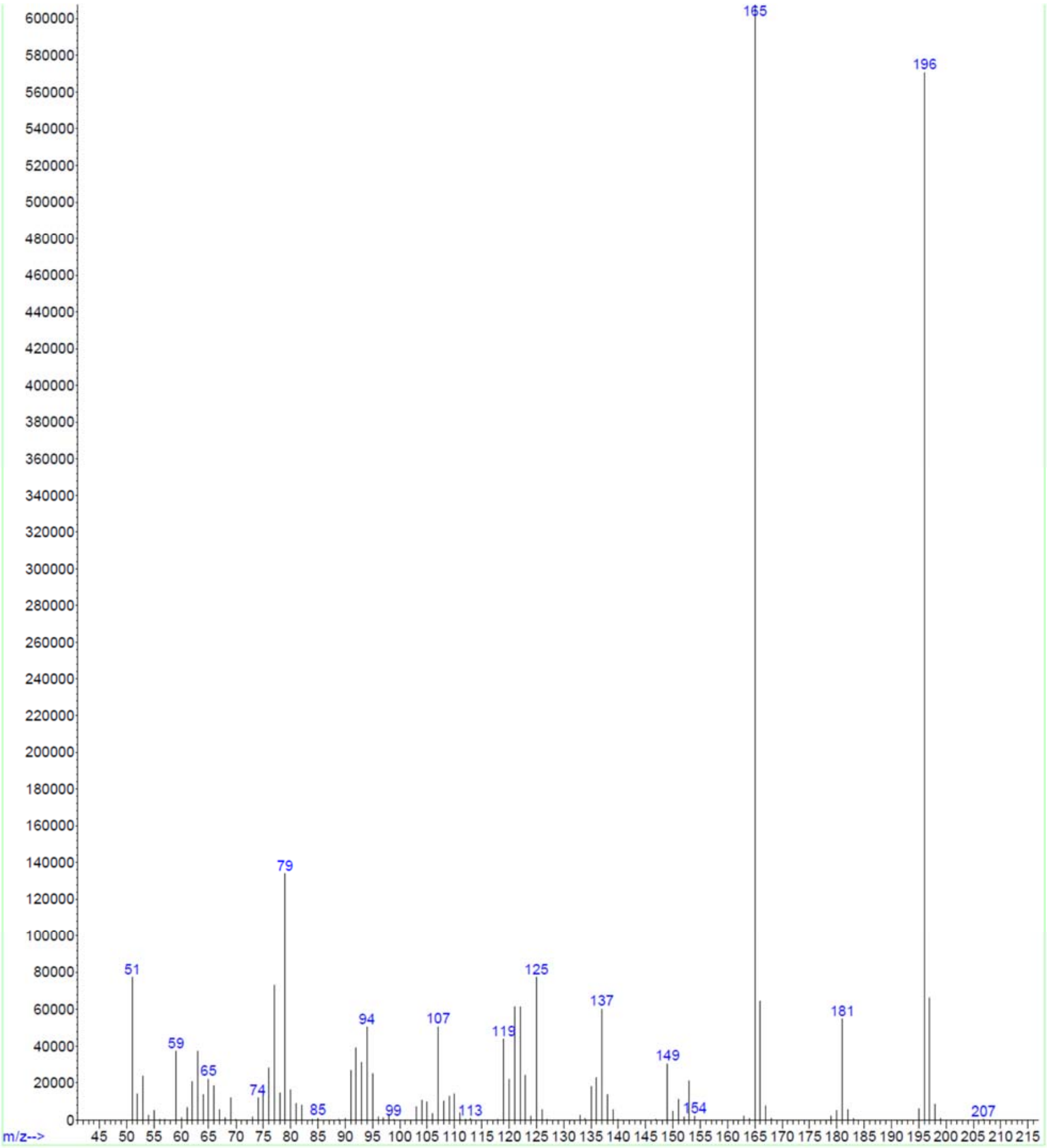

Figure S10: MS spectrum of piperonal

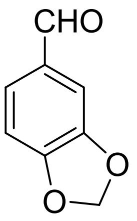

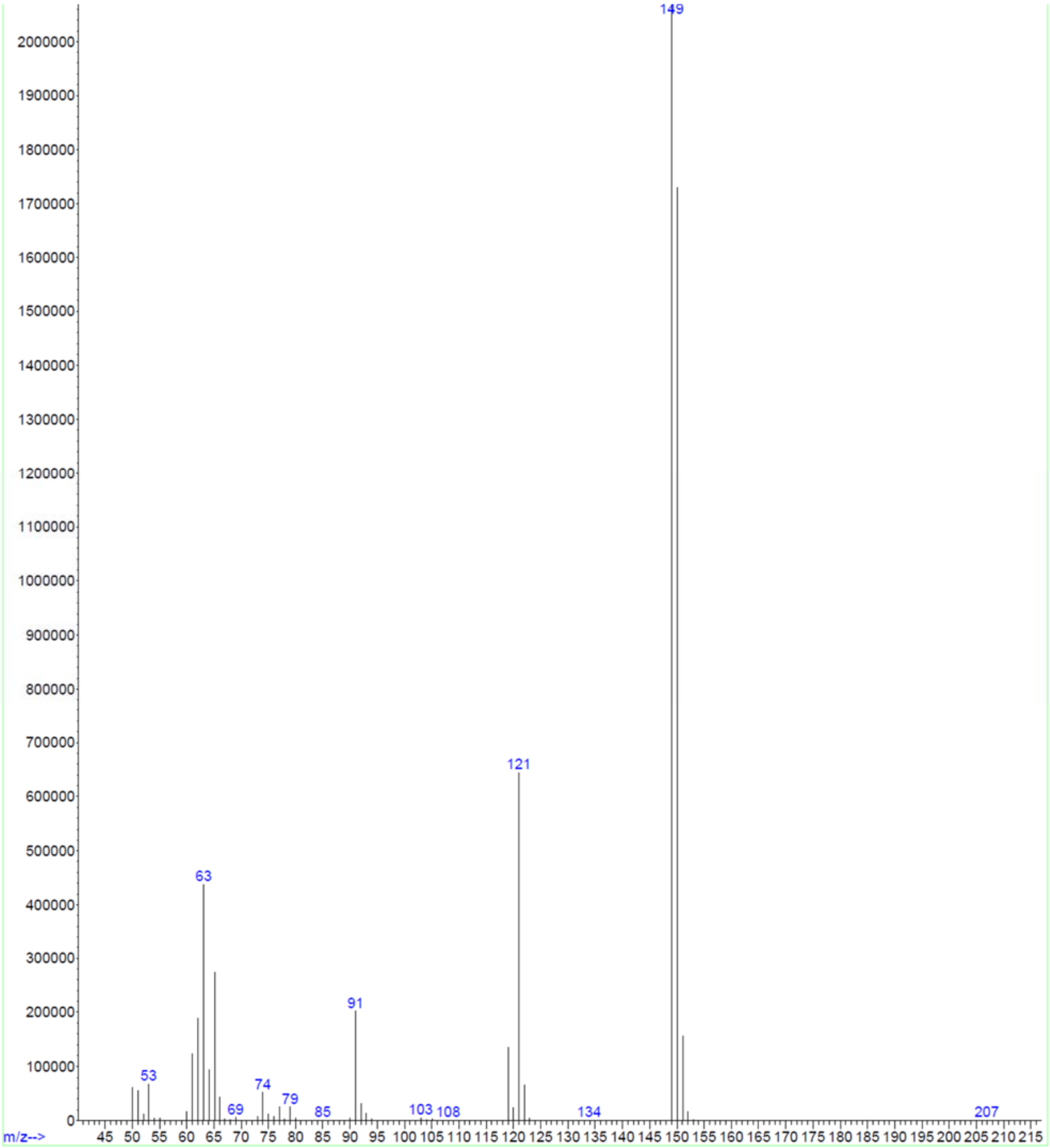

Figure S11: MS spectrum of piperonyl alcohol

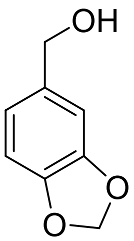

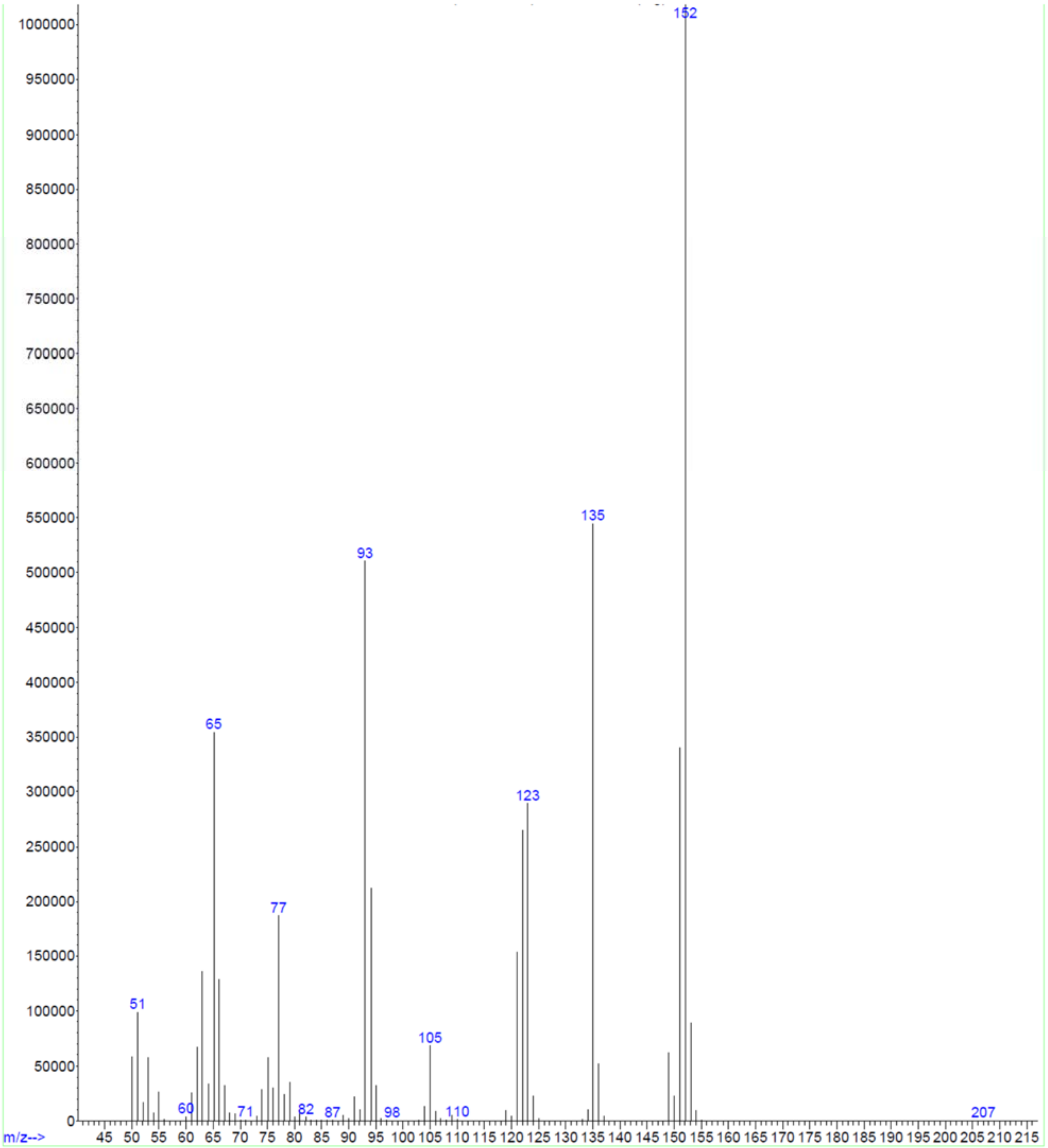

Figure S12: MS spectrum of piperonylic acid methyl ester

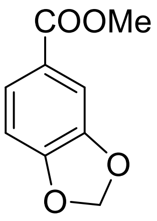

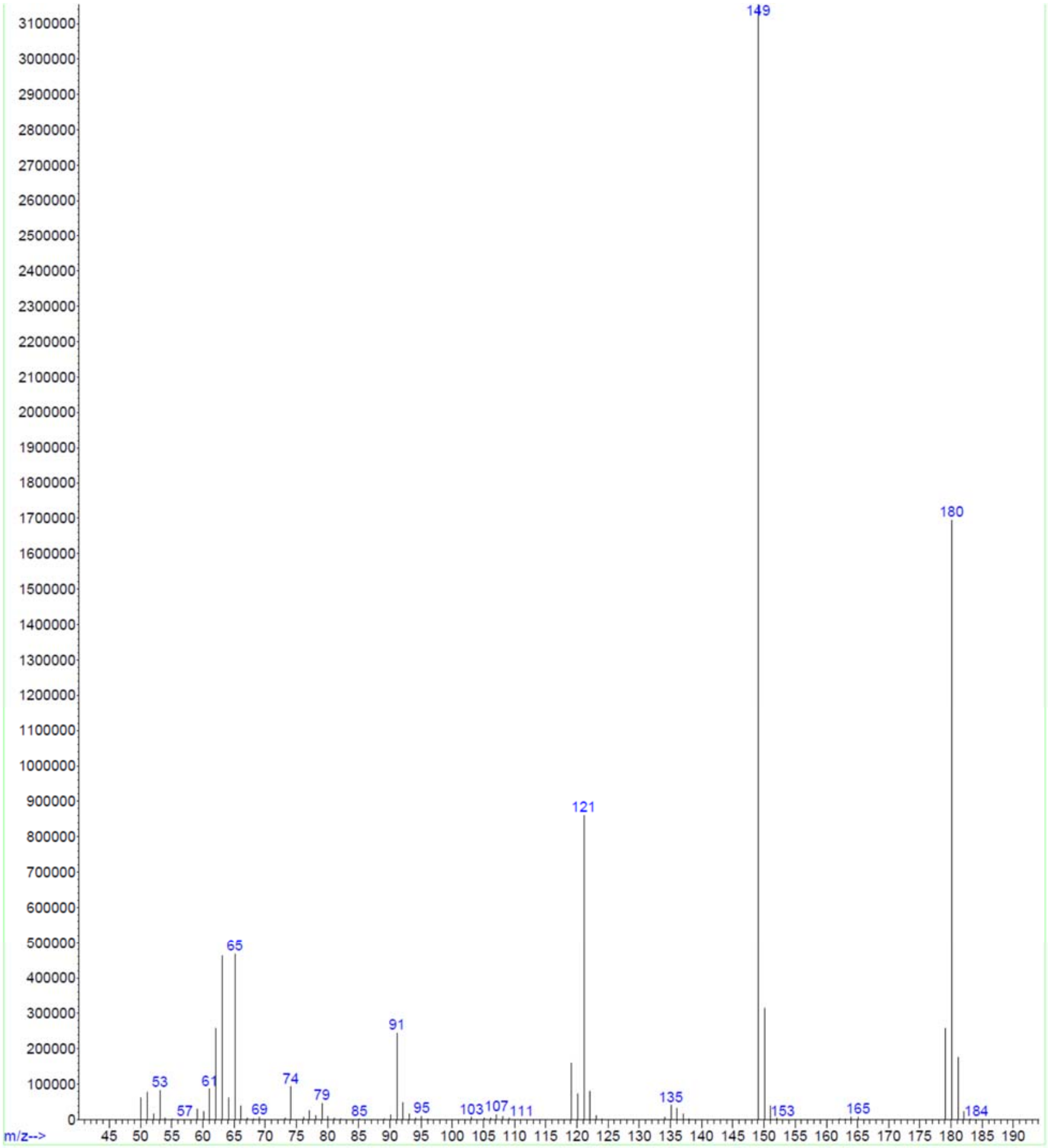

Figure S13: MS spectrum of 3,4,5-trimethoxybenzaldehyde

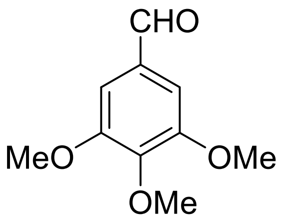

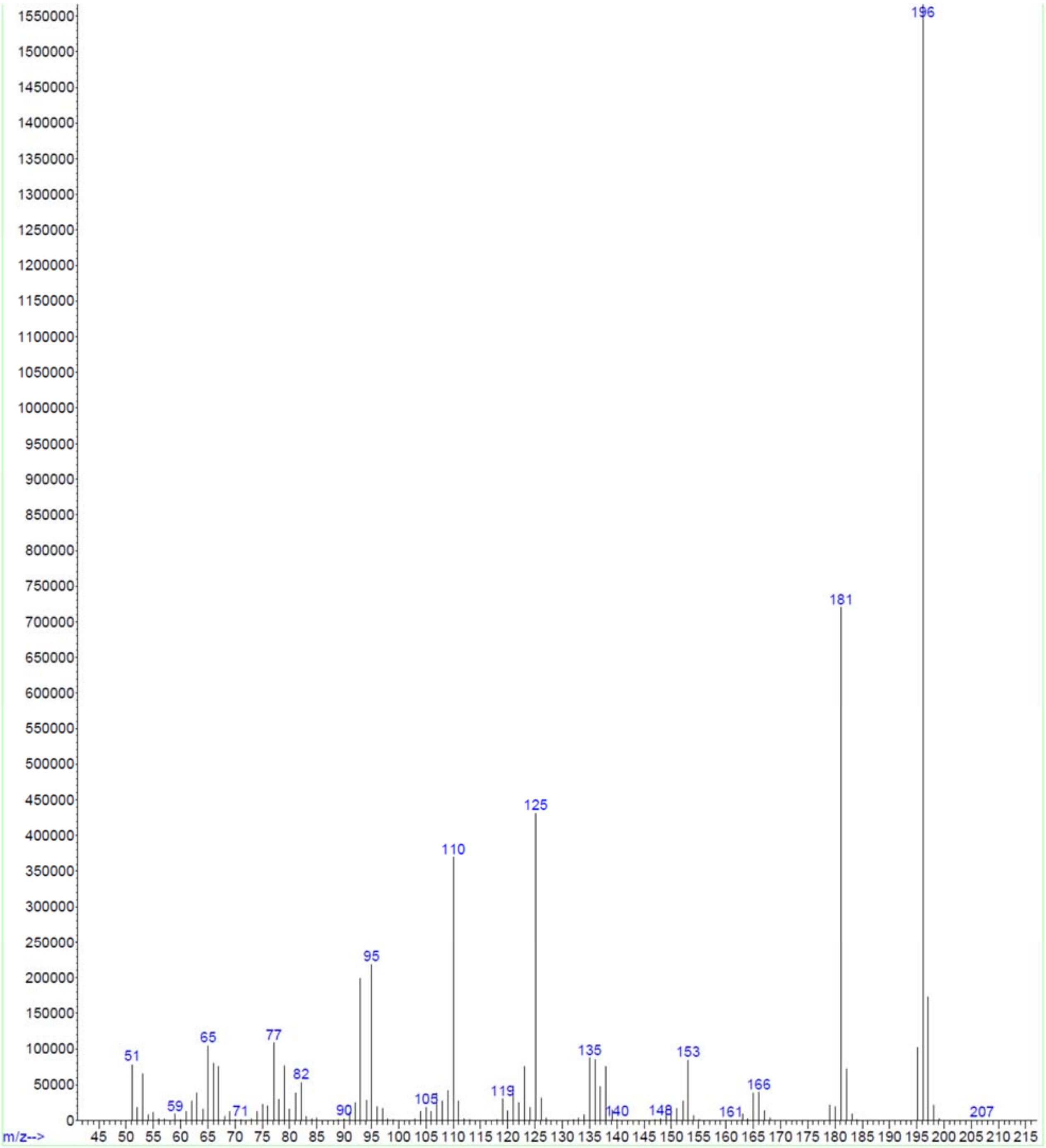

Figure S14: MS spectrum of 3,4,5-trimethoxybenzyl alcohol

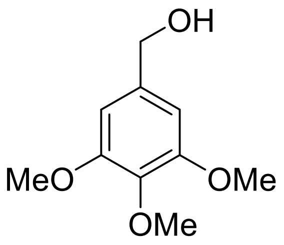

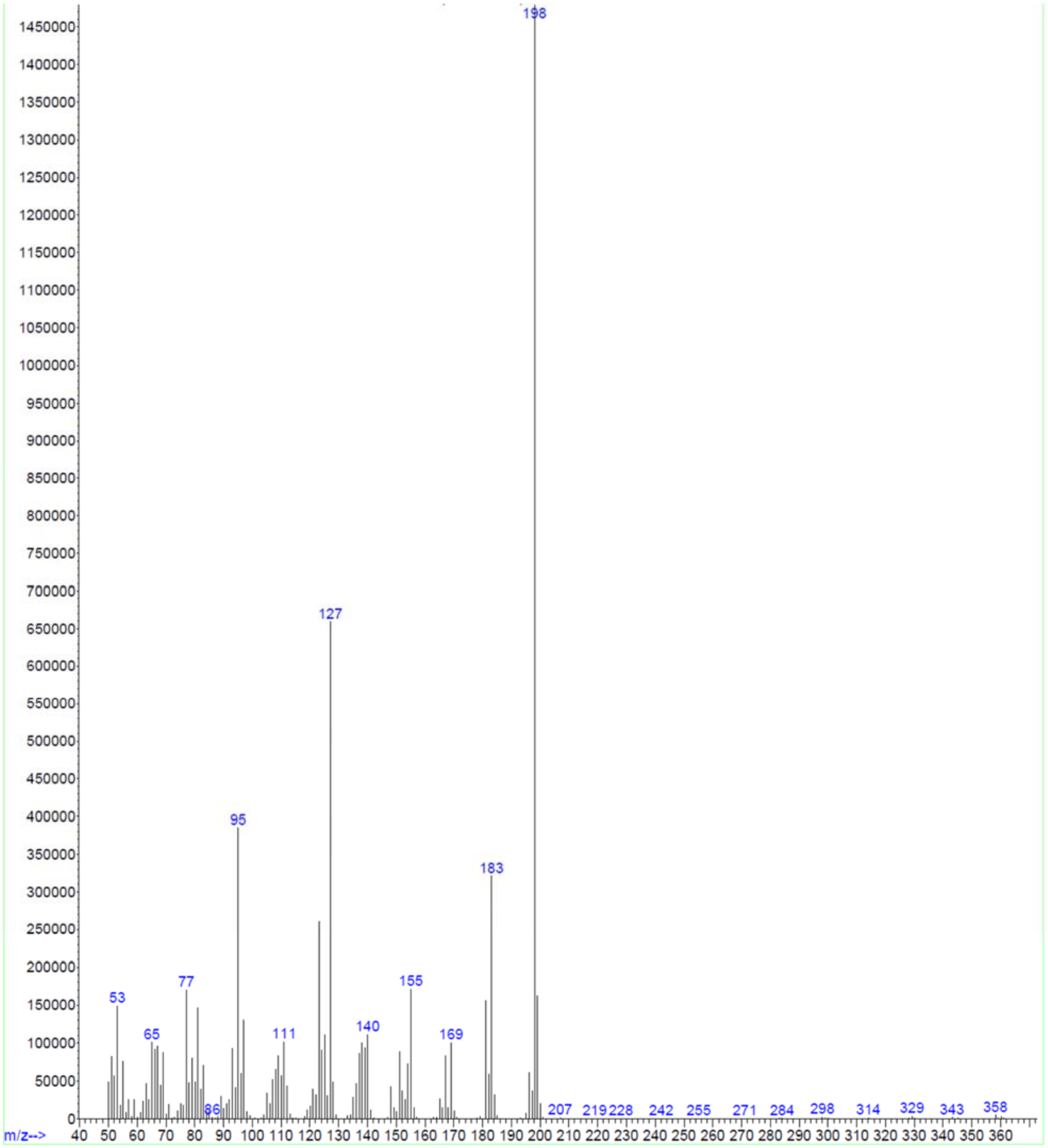

Figure S15: MS spectrum of eudesmic acid methyl ester

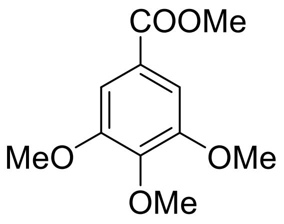

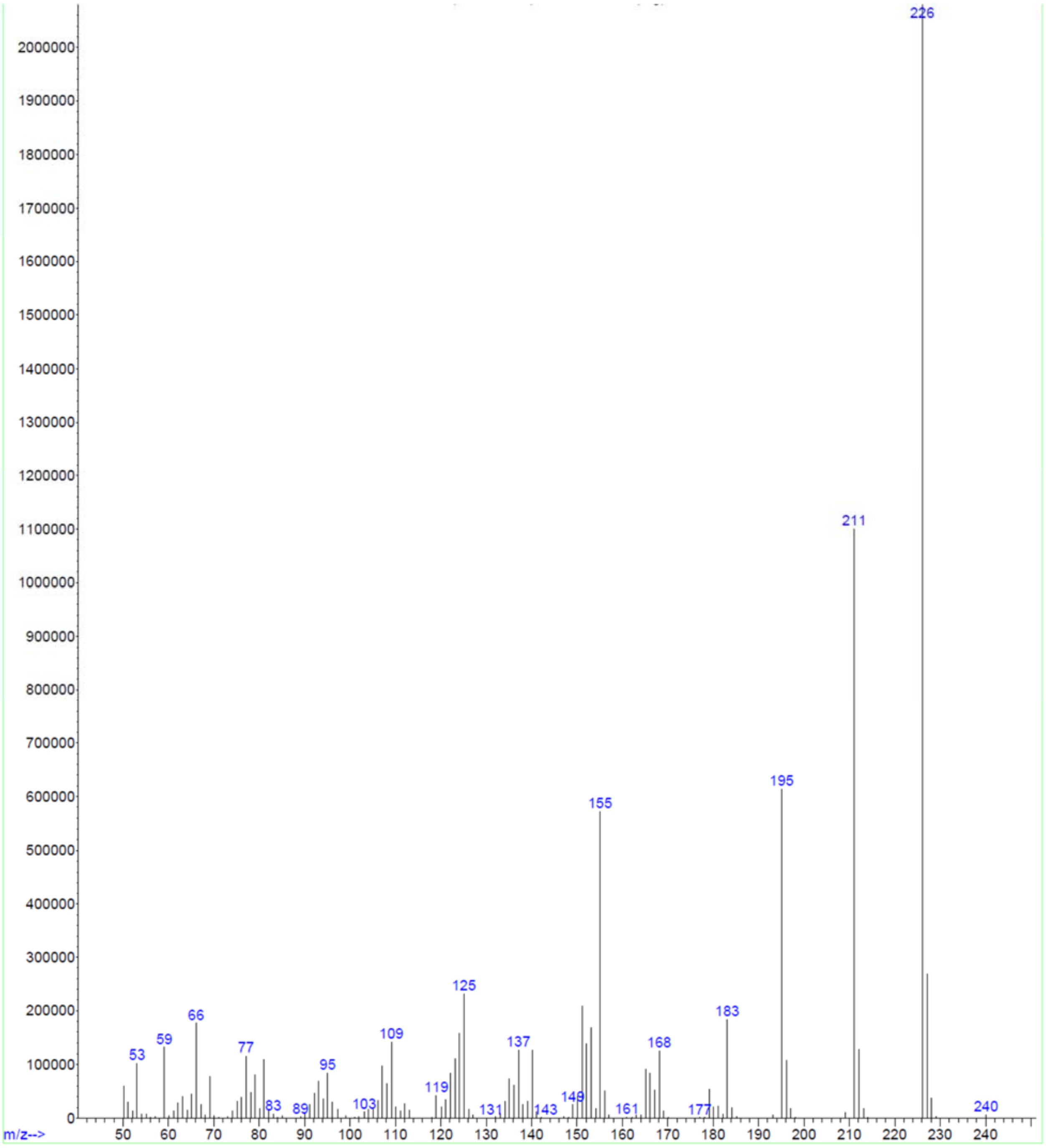

Supplement: Supplementary file 1 — Supplementary file1 (PDF 1242 KB) [file 253_2023_12872_MOESM1_ESM.pdf]
